# Comprehensive Volumetric Analysis of *Mecp2*-Null Mouse Model for Rett Syndrome by T2-Weighted 3D Magnetic Resonance Imaging

**DOI:** 10.3389/fnins.2022.885335

**Published:** 2022-05-10

**Authors:** Yuichi Akaba, Tadashi Shiohama, Yuji Komaki, Fumiko Seki, Alpen Ortug, Daisuke Sawada, Wataru Uchida, Koji Kamagata, Keigo Shimoji, Shigeki Aoki, Satoru Takahashi, Takeshi Suzuki, Jun Natsume, Emi Takahashi, Keita Tsujimura

**Affiliations:** ^1^Group of Brain Function and Development, Nagoya University Neuroscience Institute of the Graduate School of Science, Nagoya, Japan; ^2^Research Unit for Developmental Disorders, Institute for Advanced Research, Nagoya University, Nagoya, Japan; ^3^Department of Pediatrics, Asahikawa Medical University, Asahikawa, Japan; ^4^Department of Pediatrics, Chiba University Hospital, Chiba, Japan; ^5^Central Institute for Experimental Animals, Kawasaki, Japan; ^6^Department of Physiology, Keio University School of Medicine, Tokyo, Japan; ^7^Department of Radiology, Harvard Medical School, Boston, MA, United States; ^8^Athinoula A. Martinos Center for Biomedical Imaging, Massachusetts General Hospital, Boston, MA, United States; ^9^Department of Radiology, Juntendo University School of Medicine, Tokyo, Japan; ^10^Department of Diagnostic Radiology, Tokyo Metropolitan Geriatric Hospital, Tokyo, Japan; ^11^Department of Pediatrics, Nagoya University Graduate School of Medicine, Nagoya, Japan; ^12^Department of Developmental Disability Medicine, Nagoya University Graduate School of Medicine, Nagoya, Japan

**Keywords:** Rett syndrome, methyl-CpG-binding protein 2, magnetic resonance imaging, brain structure, volumetric analysis, neurodevelopmental disorder

## Abstract

Rett syndrome (RTT) is a severe progressive neurodevelopmental disorder characterized by various neurological symptoms. Almost all RTT cases are caused by mutations in the X-linked methyl-CpG-binding protein 2 (*MeCP2*) gene, and several mouse models have been established to understand the disease. However, the neuroanatomical abnormalities in each brain region of RTT mouse models have not been fully understood. Here, we investigated the global and local neuroanatomy of the *Mecp2* gene-deleted RTT model (*Mecp2*-KO) mouse brain using T2-weighted 3D magnetic resonance imaging with different morphometry to clarify the brain structural abnormalities that are involved in the pathophysiology of RTT. We found a significant reduction in global and almost all local volumes in the brain of *Mecp2*-KO mice. In addition, a detailed comparative analysis identified specific volume reductions in several brain regions in the *Mecp2*-deficient brain. Our analysis also revealed that the *Mecp2*-deficient brain shows changes in hemispheric asymmetry in several brain regions. These findings suggest that MeCP2 affects not only the whole-brain volume but also the region-specific brain structure. Our study provides a framework for neuroanatomical studies of a mouse model of RTT.

## Introduction

Rett syndrome (RTT) is a severe and progressive neurodevelopmental disorder caused by mutations in the X-linked gene encoding methyl-CpG-binding protein 2 (*MECP2*) (Amir et al., 1999). With an incidence of approximately 1:10,000 female births, RTT is one of the most common causes of severe intellectual disability in adult female (Laurvick et al., 2006). Patients with RTT show normal development up to 18 months of age. This course is followed by the loss of acquired fine and gross motor skills and the ability to engage in social interaction. In many cases, patients develop seizures, cognitive impairment, autonomic dysfunction, and stereotypic hand movements (Chahrour and Zoghbi, 2007). Autopsy studies of patients with RTT revealed a 12–34% reduction in brain weight and volume, and the effect was most pronounced in the prefrontal, posterior frontal, and anterior temporal regions, with structural abnormalities at the cellular level, such as decreased dendritic length, reduced spine density, and cell body size (Belichenko et al., 1994; Bauman et al., 1995; Armstrong, 2005). In other studies, the RTT brain showed no obvious degeneration, atrophy, or inflammation, indicating that RTT is a postnatal neurodevelopmental disorder rather than a neurodegenerative disorder (Jellinger et al., 1988; Reiss et al., 1993; Chahrour and Zoghbi, 2007). In particular, anxiety is one of the prominent symptom of the behavioral phenotype of RTT (Barnes et al., 2015). A series of studies have reported a high prevalence of anxiety and anxiety-related disorders such as fear in RTT, such as sudden mood changes, screaming episodes, inability to stop crying, and self-abuse (Sansom et al., 1993; Barnes et al., 2015; Cianfaglione et al., 2015).

To further understand the pathophysiology of RTT, several mouse models with different *Mecp2* mutations were generated in the past. *Mecp2*-knockout (*Mecp2*-KO) mice, lacking either exon 3 or both exons 3 and 4 (Chen et al., 2001; Guy et al., 2001) or carrying a truncated allele of *Mecp2* at amino acid 308 (Shahbazian et al., 2002) undergo a period of normal development, followed by severe progressive neurological phenotypes such as motor impairments, seizures, stereotypic forepaw movements, hypoactivity, and microcephaly. Female *Mecp2*± mice also had behavioral abnormalities, but with a later age of onset. Conditional *Mecp2* deletion in the brain, using the Nestin-Cre transgene, results in a phenotype similar to that observed in conventional *Mecp2*-KO mice, demonstrating that MeCP2 dysfunction in the brain is sufficient to cause the disease (Guy et al., 2001). In addition to mouse models, rat and zebrafish models that mimic *Mecp2* loss have been developed (Pietri et al., 2013; Veeraragavan et al., 2016). While the rat model has been reported to show similar developmental and behavioral abnormalities, it has been shown that zebrafish model is viable and fertile, suggesting that *Mecp2* might be more indispensable in higher organisms.

Although RTT patients and mouse models show profound neurological phenotypes, the major neuropathological changes in the brain are characterized by an overall decrease in brain size (Bauman et al., 1995; Kaufmann et al., 2000; Chen et al., 2001; Armstrong, 2005). Magnetic resonance imaging (MRI) has been used to identify specific pathological changes in the brain. MRI has the advantage of enabling non-invasive imaging, and it can be used to obtain functional and structural information of the whole brain. MRI completely covers brain volume without limiting depth penetration, and high-resolution three-dimensional (3D) imaging can be achieved accordingly (Wu and Lo, 2017). Therefore, a detailed analysis using MRI technology may reveal important insights into RTT pathology and the impacts of *MeCP2* on brain structure development when conducted in mouse models with *Mecp2* mutations. MRI studies of RTT patients have revealed volume reductions in frontal gray matter, basal ganglia, substantia nigra, midbrain, cerebellum, and brainstem area and preservation of the occipital cortex (Murakami et al., 1992; Reiss et al., 1993). Preferential reduction of the anterior frontal lobe area appears to correlate with clinical severity in patients (Carter et al., 2008). Another report of a study in which MRI was performed for children with RTT revealed decreased volumes of the cerebellum, whereas cerebral cortical volumes and subcortical gray matter volumes were preserved in the children (Shiohama et al., 2019). In addition, MRI studies using a mouse model for RTT such as *Mecp2*-null KO mice showed volume loss in many of the same areas as humans, suggesting that these models reproduce the human phenotypic gross anatomy (Saywell et al., 2006; Ward et al., 2009). However, these studies using RTT mouse models limited their analyses to certain areas and detailed volume changes throughout the Mecp2-deficient brain have not been fully investigated.

In this study, we performed a detailed whole-brain anatomical analysis of RTT mouse models using T2-weighted 3D MRI with different morphometric analysis processes. We identified Mecp2-deficient specific changes in brain structure and laterality, which are associated with the phenotypes of RTT patients and mouse models. Our study provides a framework for neuroanatomical studies of RTT mouse models.

## Materials and Methods

### Experimental Animals

All aspects of animal care and treatment were performed according to the guidelines of the Experimental Animal Care Committee of Nagoya University. *Mecp2*-KO mice (Mecp2*^tm1.1Jae^*) were generated by deleting exon 3, containing the methyl-DNA-binding domain of *Mecp2* (Chen et al., 2001), and they were obtained from Jackson Laboratories. All mice used in this study, both mutant and wild-type (WT) littermates were bred from wild-type C57BL/6J males and Mecp2*^tm1.1Jae^* heterozygous females. All mice were housed as 2–5 animals per cage and maintained on a 12-h light/dark cycle with water and food available *ad libitum*. *Mecp2*-KO male mice at the age of 6 weeks and their WT littermates were used in this study (*n* = 4 WT males, 4 *Mecp2*-KO males).

### Genotyping

After weaning, mouse genomic DNA was extracted from the tip of the tail using phenol-chloroform DNA extraction, and a polymerase chain reaction strategy was used to distinguish WT from mutant alleles using standard methodologies.

### Tissue Preparation for Magnetic Resonance Imaging

The mice were initially anesthetized with an intraperitoneal injection of a mixture of medetomidine, midazolam, and butorphanol, and then intracardially perfused as described previously (Cahill et al., 2012). Briefly, following transcardial perfusion with phosphate-buffered saline and 4% paraformaldehyde, the heads of the mice were decapitated, and their skin and lower jaw were removed accordingly.

### Magnetic Resonance Imaging Acquisition and Processing

#### Magnetic Resonance Imaging Acquisition

The MRI acquisition method has been described previously (Yano et al., 2018; Abe et al., 2019). The brains were immersed in 0.2 mM gadolinium containing PBS for 1 week. The brains were firmly fixed in an acrylic tube filled with Fluorinert (Sumitomo 3M Limited, Tokyo, Japan) to minimize the signal intensity attributable to the medium surrounding the specimen. *Ex vivo* MRI of mouse brains was performed with a 7 T Biospec 70/16 MRI scanner (Bruker Biospin GmbH, Ettlingen, Germany) equipped with actively shielded gradients at a maximum strength of 700 mT/m and a transmitting/receiving volume coil with an inner diameter of 22 mm. High-resolution anatomical images of the whole brain were acquired using a rapid acquisition with relaxation enhancement (RARE) sequence with the following parameters: effective echo time (eTE) = 20 ms, repetition time (TR) = 350 ms, RARE factor = 4, number of averages = 12, spatial resolution = 75 × 75 × 75 (μm)^3^, scan time 8 h 19 min 48 s.

#### Atlas Registration and Quantifying Anatomical Regions

Processing pipeline 1: The acquired structural T2-weighted images were registered to atlas coordinates (Lein et al., 2007; Oh et al., 2014; Hikishima et al., 2017) using the script ‘‘antsRegistrationSyN.sh’’ in Advanced Normalization Tools (ANTs) open-source software.^1^ Each brain label (575 regions in total) (Komaki et al., 2016) was obtained by applying an inverse transformation based on the registration information from the atlas coordinates to the native coordinates of the individual data (Uematsu et al., 2017; Takata et al., 2021). The individual label volume, which was automatically segmented by the ANT pipeline, was measured using the ITK-SNAP (Yushkevich et al., 2006; Seki et al., 2017).

Processing pipeline 2: The acquired structural T2-weighted images were analyzed using the Atlas Normalization Toolbox using elastiX version 2 (ANTx2) (Lein et al., 2007; Hubner et al., 2017; Koch et al., 2019)^2^ running in MATLAB (MathWorks, Natick, MA, United States) toolbox for image registration of mouse MRI data. Through the ANTx2 pipeline, MR images were processed using SPM12^3^ and non-linear warping of tissue probability maps in ELASTIX (Klein et al., 2010),^4^ and these were registered in the Allen Mouse Atlas 2017 (CCFv3) (Lein et al., 2007; Hikishima et al., 2017; Hubner et al., 2017). After checking the visual inspection of atlas registration, the estimated volumes for each anatomical region in the native space were individually calculated for each mouse.

### Evaluation of Structural Hemispheric Asymmetry

The laterality index (LI) was calculated for each mouse as [V_*L*_ − V_*R*_]/[V_*L*_ + V_*R*_] × 100, as described previously (Springer et al., 1999). V_*L*_ and V_*R*_ were the volumes for the left and right hemispheres, respectively. LIs were subsequently classified as left hemisphere dominant (LI > 20), symmetric (−20 ≤ LI ≤ +20), or right hemisphere dominant (LI < −20).

### Ethics Statement

All animal experiments were conducted under protocols approved by the Animal Experimental Committee and the recombinant DNA experiment committee of Nagoya University.

### Statistical Analysis

Statistical analysis was performed using Prism 7 (GraphPad Software, San Diego, CA, United States) and IBM SPSS statistics (IBM, Armonk, NY, United States). All data are presented as mean ± standard error of the mean (SEM). Differences between groups were examined for statistical significance using the student’s *t*-test, Welch’s *t*-test or Mann-Whitney test, followed by Bonferroni correction or false discovery rate (FDR) adjustment for multiple comparisons. Statistical significance was set at a *p*-value of < 0.05.

## Results

### *Mecp2*-Null Mice Show Reduced Body Weight and Whole-Brain Volume

**FIGURE 1:**
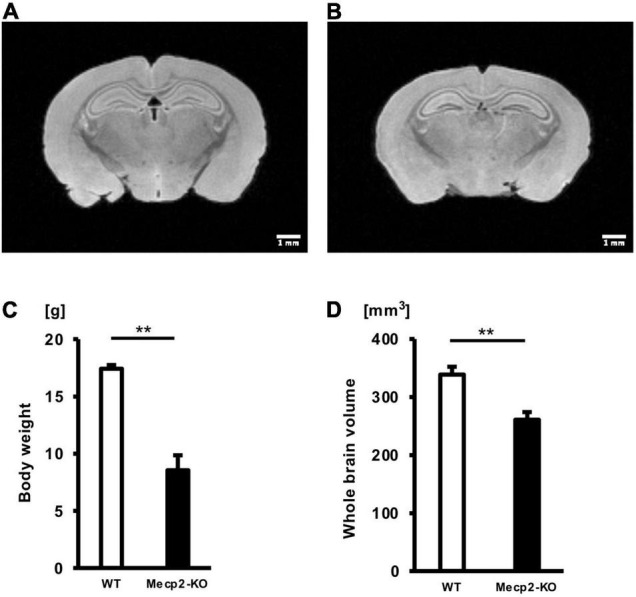
Mean body weights and whole-brain volumes for *Mecp2*-KO mice and WT mice. T2-weighted images of WT mice **(A)** and *Mecp2*-KO mice **(B)** are shown. Loss of Mecp2 leads to a drastic decrease in body weight (g) **(C)** and whole-brain volume (mm^3^) **(D)**. Statistical test: Welch’s *t*-test. *n* = 4 mice per group. **p < 0.01.

To investigate the effect of loss of the Mecp2 gene in the brain, we performed a T2-weighted MRI scan on hemizygous and matched WT controls (Figures 1A,B). There was a significant overall difference in body weight and whole-brain volume between *Mecp2*-KO mice and WT mice (Figures 1C,D and Table 1). *Mecp2*-KO mice weighted 49% as much as their WT littermates (WT 17.41 ± 0.32 g, *Mecp2*-KO 8.54 ± 1.30 g), and they had smaller whole-brain volumes (WT 338.62 ± 13.58 mm^3^, *Mecp2*-KO 260.82 ± 13.18 mm^3^).

**TABLE 1:** Results of each statistical test of Figure 1.

	Mann-Whitney *U* test	Welch’s *t*-test
Body weight	0.0286*	0.0049**
Whole brain volume	0.0286*	0.0063**

n = 4 mice per group. *p < 0.05. **p < 0.01.

### *Mecp2*-Deficient Brain Exhibits Decreases in Regional Brain Volume

**FIGURE 2:**
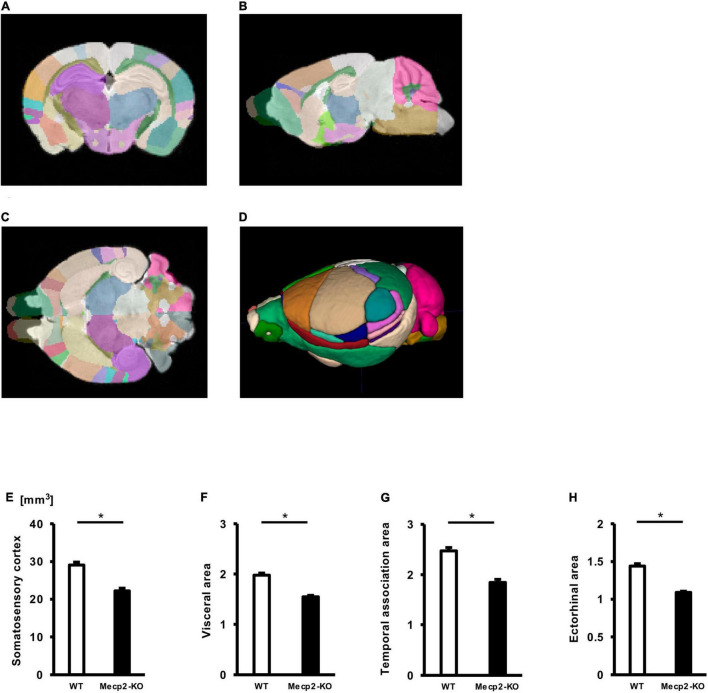
Comparison between brain region volumes of WT and *Mecp2*-KO mice obtained using volume-based morphometry. Atlas registration and quantification of anatomical regions in WT mice and *Mecp2*-KO mice were performed using volume-based morphometry **(A–D)**. Several regional brain volumes in *Mecp2*-KO mice compared with WT mice were significantly decreased, including the somatosensory cortex **(E)**, visceral area **(F)**, temporal association area **(G)**, and ectorhinal area **(H)**. Statistical test: Welch’s *t*-test. *n* = 4 mice per group. *Significant difference.

**FIGURE 3:**
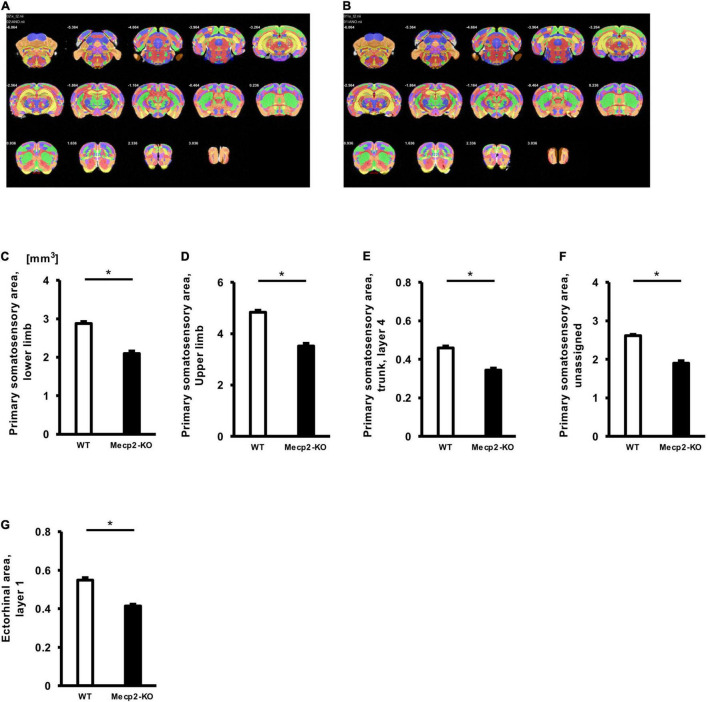
Comparison between brain region volumes of WT and *Mecp2*-KO mice obtained using detailed volume-based morphometry. Atlas registration and quantification of anatomical regions in WT mice **(A)** and *Mecp2*-KO mice **(B)** were performed using detailed volume-based morphometry. A decrease in volume was detected in brain regions such as the primary somatosensory area, lower limb **(E)**, primary somatosensory area, mouth **(C)**, primary somatosensory area, upper limb **(D)**, primary somatosensory area, trunk, layer 4 **(E)**, primary somatosensory area, unassigned **(F)**, ectorhinal area, layer 1 **(G)** in *Mecp2*-KO mice. Statistical test: Welch’s *t*-test. *n* = 4 mice per group. *Significant difference.

To evaluate potential neuroanatomical brain anomalies in *Mecp2*-KO mice, we performed an automated regional-based analysis using a mouse brain template (Processing pipeline 1). The analysis revealed that *Mecp2*-KO mice had volume reductions compared with their WT littermates across many regions of the brain, such as somatosensory area (WT 29.06 ± 0.77, *Mecp2*-KO 22.16 ± 0.71 mm^3^), visceral area (WT 1.97 ± 0.04, *Mecp2*-KO 1.54 ± 0.02 mm^3^), temporal association area (WT 2.47 ± 0.06, *Mecp2*-KO 1.84 ± 0.06 mm^3^), and ectorhinal area (WT 1.44 ± 0.03, *Mecp2*-KO 1.08 ± 0.01 mm^3^) (Figure 2, Tables 2, 3, and Supplementary Table 1). To further verify the neuroanatomical alterations in the *Mecp2*-KO brain, we conducted a more detailed analysis pipeline (Processing pipeline 2) focusing on four regions where significant volume changes were detected by processing pipeline 1. We then found volume reductions in several regions in the *Mecp2*-KO brain compared with the WT brain (Figures 3A,B, Table 4, and Supplementary Tables 2, 5). The detailed analysis revealed decreases of volume in several somatosensory areas (e.g., primary somatosensory area, lower limb (WT 2.87 ± 0.10, *Mecp2*-KO 2.09 ± 0.13 mm^3^), primary somatosensory area, upper limb (WT 4.83 ± 0.15, *Mecp2*-KO 3.51 ± 0.21 mm^3^), primary somatosensory area, trunk, layer 4 (WT 0.45 ± 0.02, *Mecp2*-KO 0.34 ± 0.01 mm^3^), and primary somatosensory area, unassigned (WT 2.61 ± 0.06, *Mecp2*-KO 1.89 ± 0.12 mm^3^) (Figures 3C–F). In addition, significant reductions were also found in ectorhinal area, layer 1 (WT 0.54 ± 0.01, *Mecp2*-KO 0.41 ± 0.01 mm^3^) (Figure 3G).

**TABLE 2:** List of significantly altered each regional brain volume in Welch’s *t*-test (Processing pipeline 1).

Calculated regional brain volume (processing pipeline 1)			
**Region**	**WT (mm^3^)**	***Mecp2*-KO (mm^3^)**	**Welch’s *t*-test**
Somatosensory area	29.06 ±0.77	22.16 ±0.71	0.0006*
Visceral area	1.97 ±0.04	1.54 ±0.02	0.0003*
Temporal association area	2.47 ±0.06	1.84 ±0.06	0.0005*
Ectorhinal area	1.44 ±0.03	1.08 ±0.01	0.0002*

n = 4 mice per group. *Significant difference (Welch’s t-test, Bonferroni correction).

**TABLE 3:** Calculated regional brain volume (processing pipeline 1; Mann Whitney *U* test, FDR adjustment).

Region	WT (mm^3^)	*Mecp2*-KO (mm^3^)	M-W *U* test *p*-value	FDR *q* = 0.2	FDR *q* = 0.1	FDR *q* = 0.05	FDR *q* = 0.025	FDR *q* = 0.01
Frontal pole, cerebral cortex	968.1975.55	735.3639.57	0.057	0.13548387	0.06774194	0.03387097	0.016935484	0.00677419
Somatomotor area	20263.18716.41	15783.81421.38	0.029	0.01290323	0.00645161	0.00322581	0.001612903	0.00064516
Somatosensory area	29064.85773.03	22169.08713.81	0.029	0.01935484	0.00967742	0.00483871	0.002419355	0.00096774
Gustatory area	1486.4650.35	1162.3735.61	0.029	0.02580645	0.01290323	0.00645161	0.003225806	0.00129032
Visceral area	1978.4941.58	1544.8328.89	0.029	0.03225806	0.01612903	0.00806452	0.004032258	0.0016129
Auditory area	4800.77147.48	3511.81179.19	0.029	0.03870968	0.01935484	0.00967742	0.00483871	0.00193548
Visual area	11082.75344.85	8352.77145.57	0.029	0.04516129	0.02258065	0.01129032	0.005645161	0.00225806
Anterior cingulate area	4735.62253.92	3574.4098.83	0.029	0.0516129	0.02580645	0.01290323	0.006451613	0.00258065
Prelimbic area	2150.66192.10	1715.3348.49	0.343	0.2	0.1	0.05	0.025	0.01
Infralimbic area	506.1152.99	401.9226.10	0.2	0.16774194	0.08387097	0.04193548	0.020967742	0.0083871
Orbital area	5150.33396.15	4159.49191.95	0.2	0.17419355	0.08709677	0.04354839	0.021774194	0.00870968
Agranular insular area	6290.56260.88	4979.97179.84	0.029	0.05806452	0.02903226	0.01451613	0.007258065	0.00290323
Retrosplenial area	8507.65122.55	6654.97250.68	0.029	0.06451613	0.03225806	0.01612903	0.008064516	0.00322581
Posterior parietal association area	2065.2862.70	1513.227.21	0.029	0.07096774	0.03548387	0.01774194	0.008870968	0.00354839
Temporal association area	2472.7065.81	1841.6764.43	0.029	0.07741935	0.03870968	0.01935484	0.009677419	0.00387097
Perirhinal area	332.546.46	246.9120.85	0.029	0.08387097	0.04193548	0.02096774	0.010483871	0.00419355
Ectorhinal area	1440.7730.04	1088.2616.13	0.029	0.09032258	0.04516129	0.02258065	0.011290323	0.00451613
Olfactory area	33444.232085.71	27225.081737.84	0.114	0.16129032	0.08064516	0.04032258	0.02016129	0.00806452
Hippocampal formation	39451.631687.02	29875.081933.83	0.029	0.09677419	0.0483871	0.02419355	0.012096774	0.00483871
Cortical subplate	5947.78196.00	4508.6774.18	0.029	0.10322581	0.0516129	0.02580645	0.012903226	0.00516129
Striatum	32923.001028.75	25027.581328.25	0.029	0.10967742	0.05483871	0.02741935	0.013709677	0.00548387
Pallidum	6500.48327.70	4959.49299.13	0.057	0.14193548	0.07096774	0.03548387	0.017741935	0.00709677
Thalamus	17926.02777.41	13727.62577.58	0.029	0.11612903	0.05806452	0.02903226	0.014516129	0.00580645
Hypothalamus	10404.74693.22	7985.79607.74	0.057	0.1483871	0.07419355	0.03709677	0.018548387	0.00741935
Midbrain	29974.902176.84	24076.931694.78	0.2	0.18064516	0.09032258	0.04516129	0.022580645	0.00903226
Hindbrain	33108.233537.82	25681.933398.90	0.2	0.18709677	0.09354839	0.04677419	0.023387097	0.00935484
Cerebellar cortex	46720.152135.70	37938.931316.11	0.057	0.15483871	0.07741935	0.03870968	0.019354839	0.00774194
Cerebellar nuclei	1789.06178.36	1442.94174.78	0.2	0.19354839	0.09677419	0.0483871	0.024193548	0.00967742
fiber tracts	25634.801457.62	19439.621318.01	0.029	0.12258065	0.06129032	0.03064516	0.015322581	0.00612903
Ventricular systems	3852.55167.97	2920.58115.77	0.029	0.12903226	0.06451613	0.03225806	0.016129032	0.00645161

*n = 4 mice per group. Significance was not detected in the case of Bonferroni correction. Red indicates significance.*

**TABLE 4:** List of significantly altered each regional brain volume in Welch’s *t*-test (Processing pipeline 2).

Calculated regional brain volume (processing pipeline 2)			

Region	WT (mm^3^)	*Mecp2*-KO (mm^3^)	Welch’s *t*-test
Primary somatosensory area, lower limb	2.87±0.10	2.09±0.13	0.0001*
Primary somatosensory area, upper limb	4.83±0.15	3.51±0.21	0.0001*
Primary somatosensory area, trunk, layer 4	0.45±0.02	0.34±0.01	0.0002*
Primary somatosensory area, unassigned	2.61±0.06	1.89±0.12	0.0003*
Ectorhinal area, layer 1	0.54±0.01	0.41±0.01	0.0002*

n = 4 mice per group. *Significant difference (Welch’s t-test, Bonferroni correction).

### The *Mecp2*-Deficient Brain Exhibits Specific Decreases in Regional Brain Volume

**FIGURE 4:**
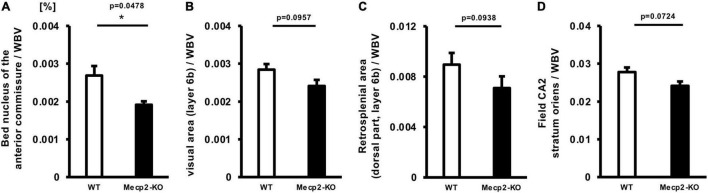
Comparison between normalized brain region volumes of WT and *Mecp2*-KO mice obtained using detailed volume-based morphometry. The ratio of each regional brain volume to the whole-brain volume was evaluated and specific regional changes, such as the bed nucleus of the anterior commissure **(A)**, posteromedial visual area (layer 6b) **(B)**, retrosplenial area **(C)**, and field CA2 stratum oriens **(D)** of *Mecp2*-KO mice were detected. Statistical test: Welch’s *t*-test. *n* = 4 mice per group. *Significant difference.

To explore brain regions that are specifically altered in *Mecp2*-deficient brains, we evaluated the ratio of each regional brain volume to the whole-brain volume and focused on brain regions that are involved in the prominent symptom of the behavioral phenotype of RTT. Then we found specific alterations in several regions of the brain of *Mecp2*-KO mice, such as the bed nucleus of the anterior commissure (WT 0.00269 ± 0.000252, *Mecp2*-KO 0.00191 ± 0.0000913%), posteromedial visual area (layer 6b) (WT 0.00284 ± 0.000148, *Mecp2*-KO 0.00241 ± 0.000161%), retrosplenial area (dorsal part, layer 6b) (WT 0.00896 ± 0.000423, *Mecp2*-KO 0.00709 ± 0.000777%), and field CA2 stratum oriens (WT 0.0278 ± 0.00121, *Mecp2*-KO 0.0241 ± 0.00115%) (Figure 4 and Table 5). These results suggest that MeCP2 deficiency also contributes to specific changes in the brain structure.

**TABLE 5:** Results of each statistical test of Figure 4.

Region	Mann-Whitney *U* test	Welch’s *t*-test
Posteromedial visual area, layer 6b/WBV	0.1143	0.0957
Retrosplenial area, dorsal part, layer 6b/WBV	0.2	0.0938
Field CA2, stratum oriens/WBV	0.1143	0.0724
Bed nucleus of the anterior commissure/WBV	0.0286*	0.0478*

*p < 0.05. n = 4 mice per group.

### Loss of *Mecp2* Affects Hemispheric Asymmetry of Brain Structure

**FIGURE 5:**
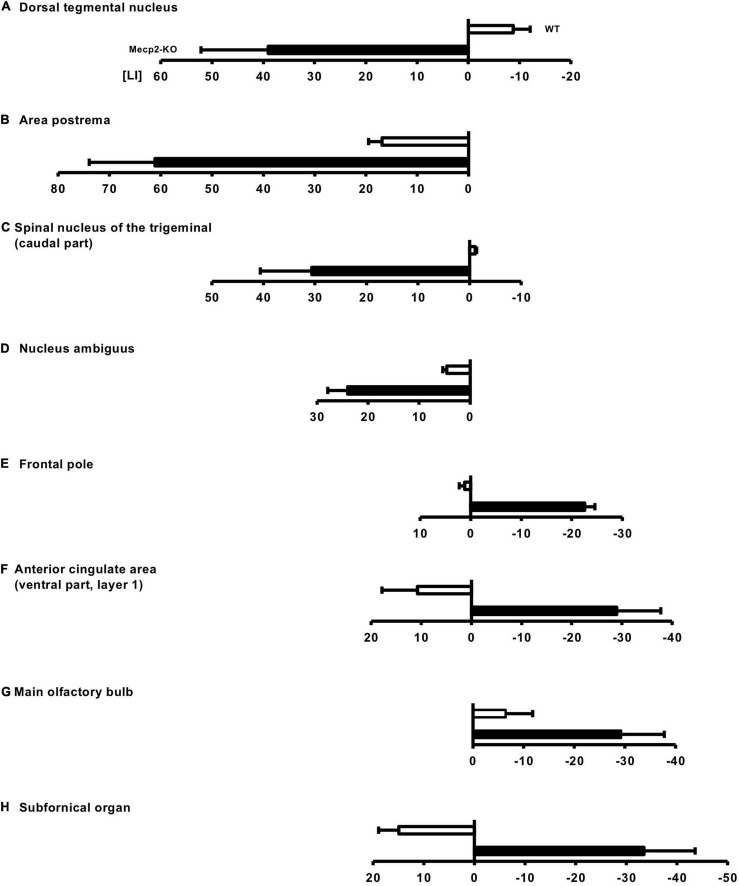
Altered laterality of the regional brain volume in *Mecp2*-KO mice. LI was calculated to evaluate the laterality of the regional brain volume in *Mecp2*-KO mice. In total, 69 regions of the left hemisphere dominant were detected in *Mecp2*-KO mice, including the dorsal tegmental nucleus **(A)**, area postrema **(B)**, spinal nucleus of the trigeminal (caudal part) **(C)**, and nucleus ambiguous **(D)**. Twenty regions of right hemisphere dominance were also detected in *Mecp2*-KO mice, including the frontal pole **(E)**, anterior cingulate area (ventral part, layer 1) **(F)**, main olfactory bulb **(G)**, and (subfornical organ) **(H)**. *n* = 4 mice per group.

Recent studies have reported that patients with neurodevelopmental and neuropsychiatric diseases show abnormal laterality of brain structures (Ribolsi et al., 2014; Okada et al., 2016; Postema et al., 2019). Since mutations in the *MeCP2* gene have been associated with a wide range of neurological disorders, we reasoned that MeCP2 affects structural asymmetry of the brain. We then investigated the laterality of the *Mecp2*-null brain. We found 69 regions of the left hemisphere dominant in volume in *Mecp2*-KO mice compared with WT mice, including the dorsal tegmental nucleus (WT LI = −8.75 ± 3.31, *Mecp2*-KO LI = 39.0 ± 13.12), area postrema (WT LI = 16.8 ± 2.62, *Mecp2*-KO LI = 61.1 ± 12.81), spinal nucleus of the trigeminal (caudal part) (WT LI = −1.08 ± 0.27, *Mecp2*-KO LI = 30.6 ± 9.99), and nucleus ambiguous (WT LI = 4.58 ± 0.79, *Mecp2*-KO LI = 24.0 ± 3.90) (Figures 5A–D and Supplementary Table 3). The analysis also revealed that the right hemisphere was dominant in 20 regions, including the frontal pole (WT LI = 1.18 ± 1.08, *Mecp2*-KO LI = −22.5 ± 2.01), anterior cingulate area (ventral part, layer 1) (WT LI = −10.7 ± 7.08, *Mecp2*-KO LI = −28.9 ± 8.80), main olfactory bulb (WT LI = −6.44 ± 5.34, *Mecp2*-KO LI = −29.2 ± 8.53), and subfornical organ (WT LI = −14.9 ± 3.99, *Mecp2*-KO LI = −33.4 ± 10.12) (Figures 5E–H and Supplementary Table 4). Together, these findings suggest that MeCP2 dysfunction influences the structural laterality of the brain.

## Discussion

In this study, we analyzed neuroanatomical measurements in T2-weighted 3D MRI of the brain of *Mecp2*-KO mice using different morphometry methods to identify the structural abnormalities that are associated with the pathophysiology of RTT. Our data showed the global and local volume reduction in the brain of *Mecp2*-KO mice compared with WT mice, and further analysis revealed the specific volume changes and laterality in several brain regions.

The overall volume reduction of the brain has consistently been found in RTT patients (Murakami et al., 1992; Reiss et al., 1993; Carter et al., 2008) and in mouse models (Saywell et al., 2006; Ward et al., 2009). These gross reductions in the RTT brain may correlate with cellular phenotypes, such as generalized reductions in neuronal soma size and dendritic arborizations (Armstrong et al., 1995). Recent studies suggest that loss of *Mecp2* induces abnormal neural stem cell (NSC)/neural progenitor cell (NPC) differentiation (Tsujimura et al., 2009, 2015; Nakashima et al., 2021) and abnormal fate specification of NSC/NPC may affect changes in brain volume. Also, since body weight of Mecp2-KO mice is decrease to about 50% of that of WT, alterations of brain volume are expected to be dependent on their body weight. However, further research on younger stage would be needed to investigate this assumption.

Furthermore, region-specific structural abnormalities that are involved in each diverse RTT phenotype including anxiety and fear have not been clarified till date. In the present study, we revealed specific changes in volume in several RTT brain regions, the bed nucleus of the anterior commissure, the posteromedial visual area (layer 6b), the retrosplenial area (dorsal part, layer 6b), and field CA2 stratum oriens, of *Mecp2*-KO mice. The ratio of the bed nucleus of the anterior commissure volume to the whole-brain volume was significantly decreased in *Mecp2*-KO mice brain, and the other three regions also showed a trend toward a decrease when compared with that of the WT brain. The bed nucleus of the anterior commissure is predominantly linked with the medial habenula and it has been implicated in the control of fear responses (Yamaguchi et al., 2013). In a previous study, ablation of the bed nucleus of the anterior commissure projection neurons selectively enhanced fear responses (Yamaguchi et al., 2013). Anxiety/fear behavior is a prominent component of the behavioral phenotype of RTT patients and it has functional consequences (Barnes et al., 2015). Fear behaviors such as “fear in unfamiliar situations” in RTT patients were reported in 46/63 cases (Coleman et al., 1988). The pathophysiology underlying anxiety/fear behavior in RTT patients is unclear and it is expected to be associated with autonomic dysfunction, as well (Robertson et al., 2006). Our data suggest that the volume reduction of bed nucleus of the anterior commissure may be directly involved in these behavioral abnormalities. The posteromedial visual area mediates visual information between the primary visual cortex and the retrosplenial cortex, which further projects to the hippocampus (Wang and Burkhalter, 2007). In humans, the retrosplenial cortex is engaged in spatial navigation and representation of familiar visual environments (Harvey et al., 2009). RTT patients have been reported to show altered visual evoked potentials (LeBlanc et al., 2015). This result indicates that *MeCP2*-deficiency causes abnormality in visual cortical processing, and alteration of the posteromedial visual area may contribute to visual processing impairment. The field CA2 of the hippocampus, particularly the vasopressin 1b receptor expressed in the hippocampus, is necessary for the regulation of social memory (Smith et al., 2016), and it promotes social aggression depending on CA2 output to the lateral septum, which is selectively enhanced immediately prior to attack (Leroy et al., 2018). RTT mouse models have deficits in long-term social learning and memory in a paradigm that has been shown to be dependent on hippocampal function of the hippocampus (Moretti et al., 2006). Externalizing behaviors, such as impulsivity, hyperactivity, aggression, self-abuse, inconsolable crying, and screaming are less frequently reported in RTT patients (Buchanan et al., 2019). A recent study showed low levels of overactivity, impulsivity, and self-abuse in RTT patients compared with a control group matched for age, sex, language, self-help skills, and intellectual ability (Cianfaglione et al., 2015). Although these facts suggest the involvement of the field CA2 in the hippocampus, *Mecp2* conditional knockout mice have also been reported to be aggressive (Fyffe et al., 2008), and several different pathophysiologies may be involved in this behavior. Since almost all of the above brain regions have not been reported as phenotype-associating regions, these findings provide novel insight into the fundamental mechanisms of pathophysiology of RTT and an interesting future study would be to evaluate the changes in the region identified in the present study in RTT patients.

This study also revealed that brain *Mecp2*-null mice exhibit alterations in structural laterality in several brain regions, such as the anterior cingulate area, subfornical organ, and dorsal tegmental nucleus (DTN). To the best of our knowledge, no studies on the alteration of structural laterality in the RTT brain have been reported so far. It is possible that these changes in laterality are associated with the pathophysiology of RTT and mouse models. A recent study showed that manipulation of the function of DTN neurons by the brainstem-acting drug cloperastine (CPS) improves breathing disorders in *Mecp2*-disrupted mice (Johnson et al., 2020). It is possible that functional alterations induced by abnormal laterality of DTN may contribute to breathing disorders in *Mecp2*-disrupted mice. The nucleus ambiguous comprises the motor neurons of the branchiogenic muscles of the 3rd–6th pharyngeal arch (CN IX, X, medullary section of XI). These form a large longitudinal rostrocaudal pars compacta of the original nucleus ambiguus. In addition, preganglionic fibers for parasympathetic innervation of the heart originate from the external part of the nucleus ambiguus and dorsal motor nucleus of the vagus nerve (dmnX) (Zandstra et al., 2021). As mentioned above, patients with RTT encounter breathing disorders. These breathing disturbances may be mechanically related to other disturbances, such as dysphagia (Ramirez et al., 2020). Dysphagia mostly arises from factors influencing chewing, such as abnormal tongue movements, usually caused by lack of movement in the central and frontal areas of the tongue, hypotonia, or hypertonia of the tongue muscles, poor posture of the cervical and thoracic spine, muscular rigidity of the shoulder girdle, hyperextension of the neck, and tongue protrusion (Mezzedimi et al., 2017). RTT patients are also associated with cardiovascular autonomic disturbances that predispose patients to cardiac arrhythmias and sudden death (Kumar et al., 2017). Immaturity of the brainstem and lack of integrative inhibition due to poor parasympathetic development have been suggested as possible explanations for autonomic dysfunction (Julu et al., 2001; Kumar et al., 2017). However, the mechanism of autonomic dysfunction in RTT is not clearly known, and altered neurotrophin signaling, serotonergic dysfunction, and substance P deficiency have been suggested as possible causes for the same (Kumar et al., 2017). We believe that abnormal laterality of the nucleus ambiguus might influence problems related to speech, shortness of breath, swallowing, and autonomic function of the heart. Only a limited number of studies have reported oral findings of Rett syndrome (Bianco and Rota, 2018). Although Rett syndrome is mostly associated with bruxism (Mahdi et al., 2021) and oral manifestations such as mouth breathing, tongue thrusting, digit/thumb sucking, high arch palate, and drooling (Ribeiro et al., 1997; Bianco and Rota, 2018; Mahdi et al., 2021), there are no reports of sensory findings or abnormalities in this area. Our findings showed a marked abnormal laterality in the caudal part (nucleus caudalis) of the spinal trigeminal nucleus, which is associated with orofacial thermal and noxious stimuli. Previously, deficits in pain sensitivity were reported in patients (Zhang et al., 2015). Zhang et al. (2014) reported that MeCP2 plays a crucial role in persistent pain sensation in mice. MeCP2 has been implicated in the early cascade of molecular steps for the initiation of pain states (Downs et al., 2010). This has also been linked to altered responses to noxious stimulation in RTT syndrome (Geranton et al., 2008; Downs et al., 2010). In addition, Zhang et al. (2015) showed that MeCP2 plays an analgesic role in both acute pain transduction and chronic pain formation in mice models by regulating the CREB-miR-132 pathway. In this light, future studies evaluating the structural laterality of the brain of patients would provide novel insights into RTT pathophysiology.

RTT was originally thought to occur exclusively in females, however, *MECP2* mutations have been identified in males presenting with classic RTT. *MECP2* mutations that cause classic RTT in females typically lead to neonatal encephalopathy and death in the first year of life in male and some of these mutations have been reported in males with classic RTT and a normal karyotype (Chahrour and Zoghbi, 2007), suggesting that *MECP2* mutations in males lead to more severe effects due to absence of normal *MECP2* and *MECP2* mutations in females exhibit moderate phenotypes compared with the case of males. Although the present our study using male hemizygote has limitations to elucidate pathology of RTT in females, the findings of this study examining the effects of *MECP2* loss on brain structure would contribute to our understanding of the brain structural pathology of female RTT. Moreover, it is very important to investigate the correlation between the severity of the symptoms and the changes in morphology for deeper understanding of the impact of *MECP2* mutation. Therefore, the future study addressing this important the issue using heterozygote female mice and other mice lines that show more severe phenotype such as a mice line generated by Bird is needed.

The mechanisms of RTT pathophysiology are still unclear, and the downstream factors of MeCP2 that are involved in the changes in brain volume have not been identified. Recent studies have shown that MeCP2 specifically promotes the processing of a subset of microRNAs (miRNAs), and miR-199a has been identified as a MeCP2-target miRNA associated with RTT pathophysiology (Tsujimura et al., 2015; Nakashima et al., 2021). These studies have also reported that genetic reduction of miR-199a in mice reproduces the phenotypes of RTT mouse models, such as microcephaly, short life, and abnormal differentiation of neural stem cells. Therefore, further research examining the global and local neuroanatomy of miR-199a-deficient mice using MRI would be an important and interesting work.

In addition, although *Mecp2*-KO mice used in this study are considered to bear a complete null mutation, they actually express a small segment of MeCP2 protein. Therefore, the possibility that the small segment of MeCP2 may affect brain structural phenotype cannot be fully ruled out, and it is needed to be addressed in future study.

In conclusion, our study performed a comprehensive volumetric analysis of *Mecp2*-null mice brain by T2-weighted 3D MRI and revealed the detailed neuroanatomical alterations and changes in structural laterality of *Mecp2*-deficient brains. These findings provide novel insights into regional alterations for RTT.

## Data Availability Statement

The original contributions presented in the study are included in the article/Supplementary Material, further inquiries can be directed to the corresponding author.

## Ethics Statement

The animal study was reviewed and approved by the Experimental Animal Care Committee of Nagoya University.

## Author Contributions

YA and KT performed the experiments and sample preparation for the MRI. YA, TSh, YK, FS, ET, and KT analyzed the data. YA, TSh, YK, DS, WU, KK, KS, SA, ST, TSu, JN, AO, ET, and KT wrote the manuscript. KT designed and supervised the entire project. All authors contributed to the article and approved the submitted version.

## Conflict of Interest

The authors declare that the research was conducted in the absence of any commercial or financial relationships that could be construed as a potential conflict of interest.

## Publisher’s Note

All claims expressed in this article are solely those of the authors and do not necessarily represent those of their affiliated organizations, or those of the publisher, the editors and the reviewers. Any product that may be evaluated in this article, or claim that may be made by its manufacturer, is not guaranteed or endorsed by the publisher.

## Footnotes

1
http://stnava.github.io/ANTs/

2
https://github.com/ChariteExpMri/antx2

3
https://www.fil.ion.ucl.ac.uk/spm/software/spm12

4
https://elastix.lumc.nl
