# Supplementary material for: Comprehensive Volumetric Analysis of Mecp2-Null Mouse Model for Rett Syndrome by T2-Weighted 3D Magnetic Resonance Imaging
Source: Front Neurosci. 2022 May 10;16:885335. doi: 10.3389/fnins.2022.885335 (PMC9127869; doi:10.3389/fnins.2022.885335)
Supplement: Supplementary file 1 [file Data_Sheet_1.pdf]

Supplementary Table 1. Each regional brain volume (Processing pipeline 1).

Calculated regional brain volume (processing pipeline 1)				
Region	WT (mm^3)		*Mecp2* -KO (mm^3)	
Frontal pole, cerebral cortex	968.19	± 75.55	735.36	± 39.57
Somatomotor area	20263.18	± 716.41	15783.81	± 421.38
Somatosensory area	29064.85	± 773.03	22169.08	± 713.81
Gustatory area	1486.46	± 50.35	1162.37	± 35.61
Visceral area	1978.49	± 41.58	1544.83	± 28.89
Auditory area	4800.77	± 147.48	3511.81	± 179.19
Visual area	11082.75	± 344.85	8352.77	± 145.57
Anterior cingulate area	4735.62	± 253.92	3574.40	± 98.83
Prelimbic area	2150.66	± 192.10	1715.33	± 48.49
Infralimbic area	506.11	± 52.99	401.92	± 26.10
Orbital area	5150.33	± 396.15	4159.49	± 191.95
Agranular insular area	6290.56	± 260.88	4979.97	± 179.84
Retrosplenial area	8507.65	± 122.55	6654.97	± 250.68
Posterior parietal association area	2065.28	± 62.70	1513.22	± 7.21
Temporal association area	2472.70	± 65.81	1841.67	± 64.43
Perirhinal area	332.54	± 6.46	246.91	± 20.85
Ectorhinal area	1440.77	± 30.04	1088.26	± 16.13
Olfactory area	33444.23	± 2085.71	27225.08	± 1737.84
Hippocampal formation	39451.63	± 1687.02	29875.08	± 1933.83
Cortical subplate	5947.78	± 196.00	4508.67	± 74.18
Striatum	32923.00	± 1028.75	25027.58	± 1328.25
Pallidum	6500.48	± 327.70	4959.49	± 299.13
Thalamus	17926.02	± 777.41	13727.62	± 577.58
Hypothalamus	10404.74	± 693.22	7985.79	± 607.74
Midbrain	29974.90	± 2176.84	24076.93	± 1694.78
Hindbrain	33108.23	± 3537.82	25681.93	± 3398.90
Cerebellar cortex	46720.15	± 2135.70	37938.93	± 1316.11
Cerebellar nuclei	1789.06	± 178.36	1442.94	± 174.78
fiber tracts	25634.80	± 1457.62	19439.62	± 1318.01
Ventricular systems	3852.55	± 167.97	2920.58	± 115.77

n = 4 mice per group.

Supplementary Table 2. Each regional brain volume (Processing pipeline 2)

Calculated regional brain volume (processing pipeline 2)		
Region	WT (mm^3)	*Mecp2* -KO (mm^3)
Cerebrum	212.313 ± 6.269	161.918 ± 7.940
Cerebral cortex	171.681 ± 5.418	131.669 ± 6.358
Cortical plate	164.567 ± 5.221	126.160 ± 6.115
Isocortex	103.285 ± 1.847	77.513 ± 2.893
Frontal pole, cerebral cortex	0.458 ± 0.021	0.332 ± 0.041
Frontal pole, layer 1	0.383 ± 0.019	0.277 ± 0.035
Frontal pole, layer 2/3	0.075 ± 0.003	0.054 ± 0.006
Somatomotor areas	19.969 ± 0.453	14.926 ± 0.498
Primary motor area	10.689 ± 0.214	7.903 ± 0.244
Primary motor area, Layer 1	1.593 ± 0.040	1.193 ± 0.035
Primary motor area, Layer 2/3	3.723 ± 0.073	2.778 ± 0.096
Primary motor area, Layer 5	3.332 ± 0.074	2.463 ± 0.083
Primary motor area, Layer 6a	1.802 ± 0.056	1.305 ± 0.034
Primary motor area, Layer 6b	0.238 ± 0.008	0.164 ± 0.005
Secondary motor area	9.281 ± 0.240	7.024 ± 0.254
Secondary motor area, layer 1	1.872 ± 0.040	1.396 ± 0.063
Secondary motor area, layer 2/3	3.111 ± 0.075	2.320 ± 0.084
Secondary motor area, layer 5	2.947 ± 0.093	2.275 ± 0.082
Secondary motor area, layer 6a	1.266 ± 0.041	0.970 ± 0.026
Secondary motor area, layer 6b	0.086 ± 0.003	0.063 ± 0.004
Somatosensory areas	32.561 ± 0.399	23.845 ± 0.879
Primary somatosensory area	27.279 ± 0.346	19.937 ± 0.699
Primary somatosensory area, nose	1.292 ± 0.022	0.960 ± 0.039
Primary somatosensory area, nose, layer 1	0.146 ± 0.005	0.109 ± 0.004
Primary somatosensory area, nose, layer 2/3	0.356 ± 0.010	0.261 ± 0.010
Primary somatosensory area, nose, layer 4	0.151 ± 0.004	0.117 ± 0.006
Primary somatosensory area, nose, layer 5	0.334 ± 0.004	0.247 ± 0.012
Primary somatosensory area, nose, layer 6a	0.250 ± 0.002	0.185 ± 0.008
Primary somatosensory area, nose, layer 6b	0.054 ± 0.001	0.041 ± 0.002
Primary somatosensory area, barrel field	9.014 ± 0.098	6.580 ± 0.269
Primary somatosensory area, barrel field, layer 1	0.862 ± 0.036	0.650 ± 0.032
Primary somatosensory area, barrel field, layer 2/3	2.628 ± 0.039	1.938 ± 0.088
Primary somatosensory area, barrel field, layer 4	1.307 ± 0.019	0.950 ± 0.036
Primary somatosensory area, barrel field, layer 5	2.191 ± 0.033	1.599 ± 0.065
Primary somatosensory area, barrel field, layer 6a	1.707 ± 0.017	1.225 ± 0.044
Primary somatosensory area, barrel field, layer 6b	0.318 ± 0.007	0.218 ± 0.007
Primary somatosensory area, lower limb	2.876 ± 0.052	2.096 ± 0.065
Primary somatosensory area, lower limb, layer 1	0.321 ± 0.007	0.238 ± 0.007
Primary somatosensory area, lower limb, layer 2/3	0.778 ± 0.017	0.579 ± 0.017
Primary somatosensory area, lower limb, layer 4	0.284 ± 0.006	0.213 ± 0.005
Primary somatosensory area, lower limb, layer 5	0.823 ± 0.021	0.604 ± 0.020
Primary somatosensory area, lower limb, layer 6a	0.559 ± 0.014	0.385 ± 0.016
Primary somatosensory area, lower limb, layer 6b	0.110 ± 0.005	0.076 ± 0.003
Primary somatosensory area, mouth	2.731 ± 0.030	1.991 ± 0.088
Primary somatosensory area, mouth, layer 1	0.328 ± 0.008	0.239 ± 0.008
Primary somatosensory area, mouth, layer 2/3	0.758 ± 0.012	0.559 ± 0.024
Primary somatosensory area, mouth, layer 4	0.299 ± 0.002	0.218 ± 0.009
Primary somatosensory area, mouth, layer 5	0.772 ± 0.012	0.566 ± 0.027
Primary somatosensory area, mouth, layer 6a	0.484 ± 0.011	0.347 ± 0.017
Primary somatosensory area, mouth, layer 6b	0.091 ± 0.001	0.063 ± 0.004
Primary somatosensory area, upper limb	4.833 ± 0.076	3.515 ± 0.106
Primary somatosensory area, upper limb, layer 1	0.528 ± 0.015	0.383 ± 0.015
Primary somatosensory area, upper limb, layer 2/3	1.406 ± 0.023	1.046 ± 0.035
Primary somatosensory area, upper limb, layer 4	0.557 ± 0.011	0.411 ± 0.014
Primary somatosensory area, upper limb, layer 5	1.345 ± 0.032	0.984 ± 0.032
Primary somatosensory area, upper limb, layer 6a	0.838 ± 0.019	0.585 ± 0.016
Primary somatosensory area, upper limb, layer 6b	0.159 ± 0.004	0.107 ± 0.004
Primary somatosensory area, trunk	3.921 ± 0.084	2.894 ± 0.112
Primary somatosensory area, trunk, layer 1	0.414 ± 0.011	0.319 ± 0.015
Primary somatosensory area, trunk, layer 2/3	1.189 ± 0.033	0.879 ± 0.029
Primary somatosensory area, trunk, layer 4	0.459 ± 0.010	0.344 ± 0.010
Primary somatosensory area, trunk, layer 5	1.021 ± 0.031	0.744 ± 0.029
Primary somatosensory area, trunk, layer 6a	0.684 ± 0.010	0.496 ± 0.026
Primary somatosensory area, trunk, layer 6b	0.155 ± 0.005	0.111 ± 0.005
Primary somatosensory area, unassigned	2.613 ± 0.034	1.900 ± 0.063
Primary somatosensory area, unassigned, layer 1	0.302 ± 0.005	0.221 ± 0.004
Primary somatosensory area, unassigned, layer 2/3	0.741 ± 0.011	0.536 ± 0.021
Primary somatosensory area, unassigned, layer 4	0.231 ± 0.006	0.173 ± 0.007
Primary somatosensory area, unassigned, layer 5	0.830 ± 0.014	0.598 ± 0.026
Primary somatosensory area, unassigned, layer 6a	0.448 ± 0.001	0.327 ± 0.010
Primary somatosensory area, unassigned, layer 6b	0.060 ± 0.001	0.044 ± 0.001
Supplemental somatosensory area	5.282 ± 0.097	3.908 ± 0.189
Supplemental somatosensory area, layer 1	0.605 ± 0.014	0.453 ± 0.020
Supplemental somatosensory area, layer 2/3	1.289 ± 0.022	0.972 ± 0.050
Supplemental somatosensory area, layer 4	0.637 ± 0.018	0.477 ± 0.027
Supplemental somatosensory area, layer 5	1.300 ± 0.025	0.965 ± 0.047
Supplemental somatosensory area, layer 6a	1.153 ± 0.025	0.826 ± 0.038
Supplemental somatosensory area, layer 6b	0.299 ± 0.008	0.214 ± 0.010
Gustatory areas	2.015 ± 0.026	1.493 ± 0.081
Gustatory areas, layer 1	0.347 ± 0.005	0.248 ± 0.013
Gustatory areas, layer 2/3	0.485 ± 0.005	0.357 ± 0.020
Gustatory areas, layer 4	0.135 ± 0.003	0.102 ± 0.005
Gustatory areas, layer 5	0.535 ± 0.009	0.398 ± 0.023
Gustatory areas, layer 6a	0.434 ± 0.005	0.330 ± 0.019
Gustatory areas, layer 6b	0.078 ± 0.002	0.059 ± 0.002
Visceral area	1.588 ± 0.017	1.187 ± 0.061
Visceral area, layer 1	0.275 ± 0.003	0.207 ± 0.009
Visceral area, layer 2/3	0.375 ± 0.004	0.282 ± 0.015
Visceral area, layer 4	0.165 ± 0.003	0.123 ± 0.007
Visceral area, layer 5	0.383 ± 0.004	0.285 ± 0.015
Visceral area, layer 6a	0.330 ± 0.006	0.243 ± 0.012
Visceral area, layer 6b	0.062 ± 0.001	0.047 ± 0.003
Auditory areas	6.423 ± 0.122	4.717 ± 0.245
Dorsal auditory area	2.067 ± 0.039	1.517 ± 0.072
Dorsal auditory area, layer 1	0.254 ± 0.004	0.190 ± 0.007
Dorsal auditory area, layer 2/3	0.596 ± 0.016	0.437 ± 0.025
Dorsal auditory area, layer 4	0.240 ± 0.009	0.178 ± 0.009
Dorsal auditory area, layer 5	0.552 ± 0.010	0.400 ± 0.019
Dorsal auditory area, layer 6a	0.320 ± 0.007	0.235 ± 0.011
Dorsal auditory area, layer 6b	0.105 ± 0.002	0.077 ± 0.004
Primary auditory area	2.174 ± 0.040	1.587 ± 0.082
Primary auditory area, layer 1	0.272 ± 0.007	0.203 ± 0.009
Primary auditory area, layer 2/3	0.587 ± 0.007	0.442 ± 0.022
Primary auditory area, layer 4	0.260 ± 0.007	0.187 ± 0.011

Primary auditory area, layer 5	0.621	± 0.011	0.451	± 0.026
Primary auditory area, layer 6a	0.339	± 0.017	0.236	± 0.016
Primary auditory area, layer 6b	0.095	± 0.003	0.070	± 0.002
Posterior auditory area	0.147	± 0.004	0.106	± 0.006
Posterior auditory area, layer 1	0.021	± 0.002	0.014	± 0.001
Posterior auditory area, layer 2/3	0.043	± 0.001	0.030	± 0.002
Posterior auditory area, layer 4	0.017	± 0.001	0.014	± 0.001
Posterior auditory area, layer 5	0.044	± 0.002	0.030	± 0.001
Posterior auditory area, layer 6a	0.017	± 0.000	0.014	± 0.001
Posterior auditory area, layer 6b	0.004	± 0.000	0.004	± 0.001
Ventral auditory area	2.035	± 0.044	1.507	± 0.086
Ventral auditory area, layer 1	0.282	± 0.002	0.221	± 0.011
Ventral auditory area, layer 2/3	0.469	± 0.015	0.348	± 0.019
Ventral auditory area, layer 4	0.234	± 0.006	0.174	± 0.012
Ventral auditory area, layer 5	0.590	± 0.011	0.437	± 0.025
Ventral auditory area, layer 6a	0.363	± 0.012	0.257	± 0.018
Ventral auditory area, layer 6b	0.097	± 0.002	0.069	± 0.005
Visual areas	9.433	± 0.316	7.155	± 0.310
Anterolateral visual area	1.547	± 0.044	1.116	± 0.058
Anterolateral visual area, layer 1	0.201	± 0.008	0.150	± 0.008
Anterolateral visual area, layer 2/3	0.433	± 0.017	0.314	± 0.017
Anterolateral visual area, layer 4	0.181	± 0.006	0.129	± 0.006
Anterolateral visual area, layer 5	0.443	± 0.010	0.320	± 0.019
Anterolateral visual area, layer 6a	0.222	± 0.005	0.158	± 0.007
Anterolateral visual area, layer 6b	0.067	± 0.002	0.045	± 0.002
Anteromedial visual area	1.400	± 0.042	1.060	± 0.049
Anteromedial visual area, layer 1	0.228	± 0.008	0.179	± 0.007
Anteromedial visual area, layer 2/3	0.369	± 0.014	0.275	± 0.012
Anteromedial visual area, layer 4	0.149	± 0.006	0.118	± 0.005
Anteromedial visual area, layer 5	0.366	± 0.009	0.280	± 0.014
Anteromedial visual area, layer 6a	0.216	± 0.005	0.157	± 0.009
Anteromedial visual area, layer 6b	0.072	± 0.003	0.052	± 0.004
Lateral visual area	0.635	± 0.028	0.480	± 0.026
Lateral visual area, layer 1	0.074	± 0.004	0.059	± 0.002
Lateral visual area, layer 2/3	0.186	± 0.007	0.142	± 0.010
Lateral visual area, layer 4	0.073	± 0.003	0.053	± 0.002
Lateral visual area, layer 5	0.169	± 0.009	0.131	± 0.007
Lateral visual area, layer 6a	0.103	± 0.006	0.072	± 0.005
Lateral visual area, layer 6b	0.029	± 0.001	0.022	± 0.002
Primary visual area	4.523	± 0.150	3.449	± 0.147
Primary visual area, layer 1	0.603	± 0.014	0.482	± 0.013
Primary visual area, layer 2/3	1.359	± 0.047	1.037	± 0.039
Primary visual area, layer 4	0.598	± 0.022	0.453	± 0.022
Primary visual area, layer 5	1.178	± 0.044	0.895	± 0.044
Primary visual area, layer 6a	0.643	± 0.022	0.479	± 0.025
Primary visual area, layer 6b	0.142	± 0.003	0.104	± 0.007
Posterolateral visual area	0.473	± 0.022	0.367	± 0.020
Posterolateral visual area, layer 1	0.064	± 0.002	0.045	± 0.003
Posterolateral visual area, layer 2/3	0.128	± 0.007	0.098	± 0.006
Posterolateral visual area, layer 4	0.054	± 0.004	0.045	± 0.002
Posterolateral visual area, layer 5	0.138	± 0.007	0.107	± 0.005
Posterolateral visual area, layer 6a	0.079	± 0.004	0.066	± 0.005
Posterolateral visual area, layer 6b	0.010	± 0.000	0.006	± 0.001
posteromedial visual area	0.856	± 0.038	0.682	± 0.019
posteromedial visual area, layer 1	0.116	± 0.013	0.103	± 0.011
posteromedial visual area, layer 2/3	0.296	± 0.012	0.235	± 0.008
posteromedial visual area, layer 4	0.088	± 0.003	0.065	± 0.003
posteromedial visual area, layer 5	0.196	± 0.005	0.155	± 0.008
posteromedial visual area, layer 6a	0.147	± 0.006	0.114	± 0.008
posteromedial visual area, layer 6b	0.013	± 0.001	0.009	± 0.001
Anterior cingulate area	4.437	± 0.154	3.316	± 0.123
Anterior cingulate area, dorsal part	2.534	± 0.089	1.935	± 0.086
Anterior cingulate area, dorsal part, layer 1	0.700	± 0.026	0.553	± 0.033
Anterior cingulate area, dorsal part, layer 2/3	0.844	± 0.038	0.636	± 0.034
Anterior cingulate area, dorsal part, layer 5	0.648	± 0.024	0.502	± 0.018
Anterior cingulate area, dorsal part, layer 6a	0.306	± 0.016	0.219	± 0.007
Anterior cingulate area, dorsal part, layer 6b	0.035	± 0.001	0.025	± 0.002
Anterior cingulate area, ventral part	1.903	± 0.077	1.380	± 0.046
Anterior cingulate area, ventral part, layer 1	0.625	± 0.019	0.482	± 0.023
Anterior cingulate area, ventral part, layer 2/3	0.630	± 0.031	0.431	± 0.007
Anterior cingulate area, ventral part, layer 5	0.429	± 0.024	0.316	± 0.021
Anterior cingulate area, ventral part, 6a	0.183	± 0.007	0.124	± 0.009
Anterior cingulate area, ventral part, 6b	0.037	± 0.002	0.027	± 0.001
Prelimbic area	1.584	± 0.038	1.275	± 0.040
Prelimbic area, layer 1	0.415	± 0.010	0.333	± 0.015
Prelimbic area, layer 2	0.189	± 0.010	0.149	± 0.005
Prelimbic area, layer 2/3	0.363	± 0.012	0.287	± 0.011
Prelimbic area, layer 5	0.380	± 0.008	0.317	± 0.011
Prelimbic area, layer 6a	0.225	± 0.008	0.180	± 0.008
Prelimbic area, layer 6b	0.011	± 0.001	0.009	± 0.001
Infralimbic area	1.478	± 0.033	1.234	± 0.045
Infralimbic area, layer 1	0.248	± 0.006	0.211	± 0.008
Infralimbic area, layer 2	0.075	± 0.003	0.063	± 0.004
Infralimbic area, layer 2/3	0.404	± 0.015	0.342	± 0.017
Infralimbic area, layer 5	0.383	± 0.014	0.317	± 0.007
Infralimbic area, layer 6a	0.305	± 0.009	0.248	± 0.010
Infralimbic area, layer 6b	0.062	± 0.003	0.053	± 0.002
Orbital area	3.721	± 0.179	3.023	± 0.129
Orbital area, lateral part	1.569	± 0.074	1.266	± 0.053
Orbital area, lateral part, layer 1	0.307	± 0.019	0.249	± 0.011
Orbital area, lateral part, layer 2/3	0.649	± 0.036	0.523	± 0.020
Orbital area, lateral part, layer 5	0.425	± 0.015	0.342	± 0.017
Orbital area, lateral part, layer 6a	0.188	± 0.005	0.152	± 0.006
Orbital area, lateral part, layer 6b	0.001	± 0.000	0.000	± 0.000
Orbital area, medial part	0.862	± 0.034	0.714	± 0.032
Orbital area, medial part, layer 1	0.205	± 0.005	0.174	± 0.010
Orbital area, medial part, layer 2	0.089	± 0.009	0.076	± 0.007
Orbital area, medial part, layer 2/3	0.256	± 0.014	0.200	± 0.009
Orbital area, medial part, layer 5	0.212	± 0.011	0.182	± 0.007
Orbital area, medial part, layer 6a	0.099	± 0.006	0.082	± 0.005
Orbital area, ventrolateral part	1.290	± 0.072	1.043	± 0.048
Orbital area, ventrolateral part, layer 1	0.279	± 0.021	0.224	± 0.015
Orbital area, ventrolateral part, layer 2/3	0.564	± 0.035	0.464	± 0.019
Orbital area, ventrolateral part, layer 5	0.231	± 0.011	0.187	± 0.008
Orbital area, ventrolateral part, layer 6a	0.215	± 0.007	0.167	± 0.008
Orbital area, ventrolateral part, layer 6b	0.002	± 0.000	0.002	± 0.000
Agranular insular area	4.319	± 0.096	3.274	± 0.187
Agranular insular area, dorsal part	2.150	± 0.057	1.624	± 0.096

Agranular insular area, dorsal part, layer 1	0.432	± 0.014	0.326	± 0.025
Agranular insular area, dorsal part, layer 2/3	0.938	± 0.027	0.715	± 0.042
Agranular insular area, dorsal part, layer 5	0.580	± 0.014	0.434	± 0.020
Agranular insular area, dorsal part, layer 6a	0.189	± 0.003	0.142	± 0.009
Agranular insular area, dorsal part, layer 6b	0.011	± 0.001	0.007	± 0.001
Agranular insular area, posterior part	1.172	± 0.019	0.892	± 0.039
Agranular insular area, posterior part, layer 1	0.343	± 0.003	0.265	± 0.013
Agranular insular area, posterior part, layer 2/3	0.334	± 0.008	0.256	± 0.014
Agranular insular area, posterior part, layer 5	0.295	± 0.005	0.225	± 0.009
Agranular insular area, posterior part, layer 6a	0.200	± 0.004	0.147	± 0.004
Agranular insular area, ventral part	0.997	± 0.025	0.757	± 0.052
Agranular insular area, ventral part, layer 1	0.172	± 0.002	0.132	± 0.011
Agranular insular area, ventral part, layer 2/3	0.401	± 0.014	0.298	± 0.022
Agranular insular area, ventral part, layer 5	0.324	± 0.007	0.249	± 0.017
Agranular insular area, ventral part, layer 6a	0.099	± 0.003	0.077	± 0.005
Agranular insular area, ventral part, layer 6b	0.001	± 0.000	0.000	± 0.000
Retrosplenial area	5.687	± 0.129	4.515	± 0.084
Retrosplenial area, lateral agranular part	0.692	± 0.025	0.541	± 0.012
Retrosplenial area, lateral agranular part, layer 1	0.121	± 0.004	0.096	± 0.002
Retrosplenial area, lateral agranular part, layer 2/3	0.213	± 0.011	0.166	± 0.003
Retrosplenial area, lateral agranular part, layer 5	0.194	± 0.006	0.152	± 0.004
Retrosplenial area, lateral agranular part, layer 6a	0.142	± 0.005	0.111	± 0.005
Retrosplenial area, lateral agranular part, layer 6b	0.021	± 0.001	0.016	± 0.001
Retrosplenial area, dorsal part	2.053	± 0.074	1.617	± 0.014
Retrosplenial area, dorsal part, layer 1	0.375	± 0.032	0.304	± 0.012
Retrosplenial area, dorsal part, layer 2/3	0.586	± 0.023	0.465	± 0.004
Retrosplenial area, dorsal part, layer 5	0.681	± 0.016	0.541	± 0.011
Retrosplenial area, dorsal part, layer 6a	0.371	± 0.005	0.282	± 0.012
Retrosplenial area, dorsal part, layer 6b	0.039	± 0.002	0.026	± 0.002
Retrosplenial area, ventral part	2.943	± 0.040	2.356	± 0.067
Retrosplenial area, ventral part, layer 1	0.606	± 0.009	0.495	± 0.016
Retrosplenial area, ventral part, layer 2	0.280	± 0.005	0.226	± 0.010
Retrosplenial area, ventral part, layer 2/3	0.550	± 0.008	0.438	± 0.012
Retrosplenial area, ventral part, layer 5	0.989	± 0.024	0.794	± 0.021
Retrosplenial area, ventral part, layer 6a	0.472	± 0.007	0.370	± 0.013
Retrosplenial area, ventral part, layer 6b	0.046	± 0.000	0.033	± 0.002
Posterior parietal association areas	2.812	± 0.064	2.081	± 0.102
Posterior parietal association areas, layer 1	0.426	± 0.011	0.316	± 0.015
Posterior parietal association areas, layer 2/3	0.756	± 0.019	0.571	± 0.031
Posterior parietal association areas, layer 4	0.261	± 0.005	0.204	± 0.011
Posterior parietal association areas, layer 5	0.787	± 0.023	0.569	± 0.023
Posterior parietal association areas, layer 6a	0.431	± 0.005	0.317	± 0.017
Posterior parietal association areas, layer 6b	0.151	± 0.007	0.104	± 0.007
Temporal association areas	2.978	± 0.074	2.224	± 0.117
Temporal association areas, layer 1	0.503	± 0.012	0.373	± 0.021
Temporal association areas, layer 2/3	0.742	± 0.016	0.554	± 0.021
Temporal association areas, layer 4	0.259	± 0.011	0.197	± 0.011
Temporal association areas, layer 5	0.896	± 0.028	0.681	± 0.044
Temporal association areas, layer 6a	0.437	± 0.012	0.317	± 0.016
Temporal association areas, layer 6b	0.141	± 0.001	0.102	± 0.008
Perirhinal area	1.163	± 0.027	0.907	± 0.038
Perirhinal area, layer 6a	0.140	± 0.004	0.111	± 0.007
Perirhinal area, layer 6b	0.057	± 0.002	0.044	± 0.003
Perirhinal area, layer 1	0.272	± 0.009	0.210	± 0.008
Perirhinal area, layer 5	0.329	± 0.006	0.252	± 0.009
Perirhinal area, layer 2/3	0.365	± 0.008	0.290	± 0.012
Ectorhinal area	2.657	± 0.076	2.010	± 0.099
Ectorhinal area/Layer 1	0.548	± 0.013	0.414	± 0.010
Ectorhinal area/Layer 2/3	0.870	± 0.027	0.651	± 0.037
Ectorhinal area/Layer 5	0.798	± 0.021	0.607	± 0.035
Ectorhinal area/Layer 6a	0.343	± 0.016	0.265	± 0.015
Ectorhinal area/Layer 6b	0.098	± 0.003	0.073	± 0.006
Olfactory areas	30.534	± 3.639	24.550	± 3.137
Main olfactory bulb	10.534	± 3.235	9.140	± 2.845
Main olfactory bulb, glomerular layer	2.494	± 0.845	2.128	± 0.700
Main olfactory bulb, granule layer	2.869	± 0.809	2.559	± 0.731
Main olfactory bulb, inner plexiform layer	0.555	± 0.165	0.484	± 0.150
Main olfactory bulb, mitral layer	0.509	± 0.153	0.428	± 0.126
Main olfactory bulb, outer plexiform layer	2.723	± 0.836	2.431	± 0.754
Accessory olfactory bulb	0.554	± 0.072	0.426	± 0.099
Accessory olfactory bulb, glomerular layer	0.111	± 0.010	0.086	± 0.018
Accessory olfactory bulb, granular layer	0.128	± 0.021	0.096	± 0.026
Accessory olfactory bulb, mitral layer	0.109	± 0.014	0.084	± 0.018
Anterior olfactory nucleus	3.507	± 0.323	2.918	± 0.172
Anterior olfactory nucleus, dorsal part	0.237	± 0.018	0.194	± 0.011
Anterior olfactory nucleus, external part	0.230	± 0.028	0.189	± 0.024
Anterior olfactory nucleus, lateral part	0.548	± 0.062	0.451	± 0.034
Anterior olfactory nucleus, medial part	0.576	± 0.044	0.476	± 0.034
Anterior olfactory nucleus, posteroventral part	0.546	± 0.057	0.442	± 0.028
Anterior olfactory nucleus, layer 1	1.060	± 0.102	0.864	± 0.048
Taenia tecta	1.488	± 0.073	1.186	± 0.073
Taenia tecta, dorsal part	1.097	± 0.042	0.876	± 0.052
Taenia tecta, dorsal part, layer 1	0.360	± 0.020	0.286	± 0.020
Taenia tecta, dorsal part, layer 2	0.375	± 0.017	0.301	± 0.017
Taenia tecta, dorsal part, layer 3	0.274	± 0.010	0.217	± 0.014
Taenia tecta, dorsal part, layer 4	0.089	± 0.001	0.071	± 0.002
Taenia tecta, ventral part	0.390	± 0.033	0.311	± 0.022
Taenia tecta, ventral part, layer 1	0.197	± 0.017	0.161	± 0.012
Taenia tecta, ventral part, layer 2	0.130	± 0.014	0.101	± 0.008
Taenia tecta, ventral part, layer 3	0.064	± 0.002	0.049	± 0.003
Dorsal peduncular area	0.270	± 0.007	0.224	± 0.008
Dorsal peduncular area, layer 1	0.086	± 0.002	0.072	± 0.002
Dorsal peduncular area, layer 2/3	0.085	± 0.004	0.069	± 0.004
Dorsal peduncular area, layer 5	0.069	± 0.002	0.062	± 0.003
Dorsal peduncular area, layer 6a	0.030	± 0.001	0.021	± 0.001
Piriform area	11.071	± 0.313	8.395	± 0.587
Piriform area, molecular layer	4.141	± 0.123	3.072	± 0.238
Piriform area, pyramidal layer	3.140	± 0.092	2.391	± 0.173
Piriform area, polymorph layer	3.790	± 0.099	2.932	± 0.179
Nucleus of the lateral olfactory tract	0.344	± 0.010	0.225	± 0.053
Nucleus of the lateral olfactory tract, molecular layer	0.144	± 0.005	0.096	± 0.024
Nucleus of the lateral olfactory tract, pyramidal layer	0.127	± 0.006	0.077	± 0.017
Nucleus of the lateral olfactory tract, layer 3	0.073	± 0.002	0.052	± 0.013
Cortical amygdalar area	2.104	± 0.112	1.584	± 0.187
Cortical amygdalar area, anterior part	0.481	± 0.020	0.326	± 0.071
Cortical amygdalar area, anterior part, layer 1	0.228	± 0.012	0.154	± 0.036
Cortical amygdalar area, anterior part, layer 2	0.253	± 0.010	0.173	± 0.035
Cortical amygdalar area, posterior part	1.623	± 0.092	1.258	± 0.119

Cortical amygdalar area, posterior part, lateral zone	0.811	± 0.044	0.607	± 0.070
Cortical amygdalar area, posterior part, lateral zone, layer 1	0.221	± 0.013	0.160	± 0.028
Cortical amygdalar area, posterior part, lateral zone, layer 2	0.260	± 0.013	0.190	± 0.023
Cortical amygdalar area, posterior part, lateral zone, layer 3	0.331	± 0.019	0.257	± 0.020
Cortical amygdalar area, posterior part, medial zone	0.812	± 0.049	0.650	± 0.052
Cortical amygdalar area, posterior part, medial zone, layer 1	0.179	± 0.010	0.140	± 0.016
Cortical amygdalar area, posterior part, medial zone, layer 2	0.215	± 0.010	0.171	± 0.011
Cortical amygdalar area, posterior part, medial zone, layer 3	0.418	± 0.029	0.339	± 0.025
Piriform-amygdalar area	0.553	± 0.022	0.411	± 0.055
Piriform-amygdalar area, molecular layer	0.224	± 0.009	0.160	± 0.027
Piriform-amygdalar area, pyramidal layer	0.139	± 0.005	0.106	± 0.014
Piriform-amygdalar area, polymorph layer	0.189	± 0.009	0.145	± 0.014
Postpiriform transition area	0.930	± 0.050	0.754	± 0.035
Postpiriform transition area, layers 1	0.333	± 0.027	0.269	± 0.014
Postpiriform transition area, layers 2	0.211	± 0.007	0.171	± 0.007
Postpiriform transition area, layers 3	0.387	± 0.018	0.314	± 0.015
Hippocampal formation	31.827	± 0.994	24.955	± 0.906
Hippocampal region	15.831	± 0.362	12.346	± 0.396
Ammon's horn	10.976	± 0.199	8.502	± 0.276
Field CA1	6.416	± 0.124	4.934	± 0.201
Field CA1, stratum lacunosum-moleculare	1.705	± 0.042	1.339	± 0.075
Field CA1, stratum oriens	1.252	± 0.017	0.944	± 0.028
Field CA1, pyramidal layer	1.419	± 0.024	1.081	± 0.036
Field CA1, stratum radiatum	2.040	± 0.045	1.570	± 0.076
Field CA2	0.455	± 0.009	0.343	± 0.015
Field CA2, stratum lacunosum-moleculare	0.113	± 0.005	0.087	± 0.003
Field CA2, stratum oriens	0.122	± 0.002	0.088	± 0.005
Field CA2, pyramidal layer	0.080	± 0.002	0.061	± 0.003
Field CA2, stratum radiatum	0.140	± 0.003	0.107	± 0.007
Field CA3	4.105	± 0.073	3.225	± 0.073
Field CA3, stratum lacunosum-moleculare	0.053	± 0.001	0.047	± 0.005
Field CA3, stratum lucidum	0.267	± 0.006	0.213	± 0.007
Field CA3, stratum oriens	1.381	± 0.013	1.080	± 0.024
Field CA3, pyramidal layer	1.202	± 0.033	0.948	± 0.024
Field CA3, stratum radiatum	1.200	± 0.029	0.937	± 0.025
Dentate gyrus	4.782	± 0.188	3.786	± 0.139
Dentate gyrus, molecular layer	3.123	± 0.120	2.463	± 0.095
Dentate gyrus, polymorph layer	0.578	± 0.021	0.456	± 0.013
Dentate gyrus, granule cell layer	1.081	± 0.050	0.866	± 0.032
Fasciola cinerea	0.048	± 0.002	0.035	± 0.002
Induseum griseum	0.025	± 0.003	0.023	± 0.001
Retrohippocampal region	15.996	± 0.656	12.609	± 0.585
Entorhinal area	9.429	± 0.401	7.554	± 0.300
Entorhinal area, lateral part	6.699	± 0.247	5.435	± 0.170
Entorhinal area, lateral part, layer 1	1.359	± 0.057	1.128	± 0.041
Entorhinal area, lateral part, layer 2	0.047	± 0.002	0.038	± 0.002
Entorhinal area, lateral part, layer 2/3	0.141	± 0.003	0.109	± 0.004
Entorhinal area, lateral part, layer 2a	0.738	± 0.035	0.601	± 0.016
Entorhinal area, lateral part, layer 2b	0.750	± 0.038	0.622	± 0.021
Entorhinal area, lateral part, layer 3	1.025	± 0.039	0.841	± 0.025
Entorhinal area, lateral part, layer 4	0.423	± 0.013	0.341	± 0.007
Entorhinal area, lateral part, layer 4/5	0.160	± 0.002	0.127	± 0.006
Entorhinal area, lateral part, layer 5	0.986	± 0.030	0.778	± 0.028
Entorhinal area, lateral part, layer 6a	0.935	± 0.034	0.739	± 0.029
Entorhinal area, lateral part, layer 6b	0.136	± 0.005	0.110	± 0.005
Entorhinal area, medial part, dorsal zone	2.536	± 0.145	1.954	± 0.120
Entorhinal area, medial part, dorsal zone, layer 1	0.701	± 0.051	0.536	± 0.029
Entorhinal area, medial part, dorsal zone, layer 2	0.708	± 0.043	0.547	± 0.036
Entorhinal area, medial part, dorsal zone, layer 2a	0.015	± 0.001	0.010	± 0.002
Entorhinal area, medial part, dorsal zone, layer 2b	0.011	± 0.001	0.010	± 0.001
Entorhinal area, medial part, dorsal zone, layer 3	0.563	± 0.026	0.439	± 0.030
Entorhinal area, medial part, dorsal zone, layer 4	0.079	± 0.003	0.059	± 0.004
Entorhinal area, medial part, dorsal zone, layer 5	0.168	± 0.012	0.122	± 0.008
Entorhinal area, medial part, dorsal zone, layer 6	0.291	± 0.012	0.231	± 0.013
Entorhinal area, medial part, ventral zone	0.193	± 0.021	0.165	± 0.012
Entorhinal area, medial part, ventral zone, layer 1	0.088	± 0.011	0.077	± 0.005
Entorhinal area, medial part, ventral zone, layer 2	0.086	± 0.010	0.073	± 0.005
Entorhinal area, medial part, ventral zone, layer 3	0.018	± 0.001	0.015	± 0.002
Entorhinal area, medial part, ventral zone, layer 5/6	0.001	± 0.000	0.001	± 0.000
Parasubiculum	0.892	± 0.070	0.679	± 0.056
Parasubiculum, layer 1	0.155	± 0.017	0.127	± 0.009
Parasubiculum, layer 2	0.499	± 0.043	0.372	± 0.035
Parasubiculum, layer 3	0.239	± 0.010	0.180	± 0.012
Postsubiculum	0.618	± 0.019	0.462	± 0.030
Postsubiculum, layer 1	0.163	± 0.006	0.126	± 0.008
Postsubiculum, layer 2	0.239	± 0.009	0.179	± 0.010
Postsubiculum, layer 3	0.216	± 0.005	0.157	± 0.013
Presubiculum	0.981	± 0.049	0.763	± 0.054
Presubiculum, layer 1	0.268	± 0.017	0.210	± 0.017
Presubiculum, layer 2	0.447	± 0.024	0.353	± 0.024
Presubiculum, layer 3	0.266	± 0.008	0.200	± 0.015
Subiculum	4.076	± 0.131	3.150	± 0.164
Subiculum, dorsal part	0.984	± 0.013	0.740	± 0.030
Subiculum, dorsal part, molecular layer	0.175	± 0.005	0.134	± 0.012
Subiculum, dorsal part, pyramidal layer	0.619	± 0.007	0.463	± 0.016
Subiculum, dorsal part, stratum radiatum	0.190	± 0.004	0.143	± 0.005
Subiculum, ventral part	3.092	± 0.125	2.411	± 0.144
Subiculum, ventral part, molecular layer	0.605	± 0.024	0.479	± 0.031
Subiculum, ventral part, pyramidal layer	2.091	± 0.083	1.607	± 0.095
Subiculum, ventral part, stratum radiatum	0.397	± 0.018	0.325	± 0.018
Cortical subplate	6.072	± 0.185	4.707	± 0.220
Claustrum	0.891	± 0.015	0.668	± 0.030
Endopiriform nucleus	2.247	± 0.059	1.739	± 0.075
Endopiriform nucleus, dorsal part	1.801	± 0.042	1.398	± 0.060
Endopiriform nucleus, ventral part	0.446	± 0.018	0.341	± 0.015
Lateral amygdalar nucleus	0.544	± 0.018	0.409	± 0.014
Basolateral amygdalar nucleus	1.231	± 0.050	0.966	± 0.040
Basolateral amygdalar nucleus, anterior part	0.650	± 0.023	0.513	± 0.022
Basolateral amygdalar nucleus, posterior part	0.380	± 0.016	0.289	± 0.017
Basolateral amygdalar nucleus, ventral part	0.201	± 0.012	0.165	± 0.009
Basomedial amygdalar nucleus	0.897	± 0.026	0.707	± 0.053
Basomedial amygdalar nucleus, anterior part	0.264	± 0.005	0.202	± 0.021
Basomedial amygdalar nucleus, posterior part	0.632	± 0.022	0.505	± 0.034
Posterior amygdalar nucleus	0.263	± 0.022	0.218	± 0.014
Cerebral nuclei	41.674	± 1.010	31.052	± 1.676
Striatum	34.028	± 0.772	25.255	± 1.344
Striatum dorsal region	20.233	± 0.227	15.012	± 0.575
Caudoputamen	20.233	± 0.227	15.012	± 0.575

Striatum ventral region	7.907	± 0.451	5.856	± 0.559
Nucleus accumbens	4.256	± 0.222	3.230	± 0.237
Fundus of striatum	0.319	± 0.008	0.241	± 0.019
Olfactory tubercle	3.332	± 0.233	2.385	± 0.309
Islands of Calleja	0.215	± 0.017	0.164	± 0.020
Major island of Calleja	0.042	± 0.003	0.029	± 0.003
Olfactory tubercle, molecular layer	1.116	± 0.075	0.797	± 0.101
Olfactory tubercle, pyramidal layer	1.067	± 0.080	0.762	± 0.106
Olfactory tubercle, polymorph layer	0.892	± 0.067	0.634	± 0.086
Lateral septal complex	2.894	± 0.021	2.130	± 0.071
Lateral septal nucleus	2.604	± 0.021	1.911	± 0.062
Lateral septal nucleus, caudal (caudodorsal) part	0.518	± 0.019	0.367	± 0.031
Lateral septal nucleus, rostral (rostroventral) part	1.995	± 0.033	1.479	± 0.047
Lateral septal nucleus, ventral part	0.091	± 0.002	0.065	± 0.003
Septofimbrial nucleus	0.220	± 0.002	0.161	± 0.007
Septohippocampal nucleus	0.070	± 0.001	0.058	± 0.005
Striatum-like amygdalar nuclei	3.254	± 0.098	2.461	± 0.187
Anterior amygdalar area	0.502	± 0.016	0.361	± 0.052
Bed nucleus of the accessory olfactory tract	0.057	± 0.004	0.038	± 0.006
Central amygdalar nucleus	1.008	± 0.031	0.785	± 0.037
Central amygdalar nucleus, capsular part	0.396	± 0.009	0.301	± 0.015
Central amygdalar nucleus, lateral part	0.169	± 0.007	0.134	± 0.008
Central amygdalar nucleus, medial part	0.442	± 0.016	0.351	± 0.015
Intercalated amygdalar nucleus	0.166	± 0.000	0.126	± 0.010
Medial amygdalar nucleus	1.262	± 0.039	0.948	± 0.080
Medial amygdalar nucleus, anterodorsal part	0.566	± 0.013	0.435	± 0.032
Medial amygdalar nucleus, anteroventral part	0.337	± 0.011	0.231	± 0.036
Medial amygdalar nucleus, posterodorsal part	0.298	± 0.015	0.235	± 0.010
Medial amygdalar nucleus, posterodorsal part, sublayer a	0.120	± 0.005	0.092	± 0.005
Medial amygdalar nucleus, posterodorsal part, sublayer b	0.074	± 0.005	0.061	± 0.004
Medial amygdalar nucleus, posterodorsal part, sublayer c	0.104	± 0.004	0.082	± 0.002
Medial amygdalar nucleus, posteroventral part	0.060	± 0.003	0.047	± 0.005
Pallidum	7.646	± 0.240	5.797	± 0.342
Pallidum, dorsal region	1.540	± 0.049	1.192	± 0.057
Globus pallidus, external segment	1.286	± 0.039	0.993	± 0.046
Globus pallidus, internal segment	0.254	± 0.010	0.199	± 0.011
Pallidum, ventral region	3.111	± 0.117	2.345	± 0.198
Substantia innominata	2.789	± 0.108	2.112	± 0.164
Magnocellular nucleus	0.323	± 0.011	0.233	± 0.036
Pallidum, medial region	1.683	± 0.042	1.295	± 0.072
Medial septal complex	1.550	± 0.042	1.193	± 0.071
Medial septal nucleus	0.566	± 0.015	0.414	± 0.021
Diagonal band nucleus	0.984	± 0.031	0.779	± 0.051
Triangular nucleus of septum	0.133	± 0.004	0.102	± 0.006
Pallidum, caudal region	1.311	± 0.039	0.965	± 0.037
Bed nuclei of the stria terminalis	1.299	± 0.038	0.958	± 0.037
Bed nuclei of the stria terminalis, anterior division	0.808	± 0.031	0.595	± 0.026
Bed nuclei of the stria terminalis, anterior division, anterolateral area	0.238	± 0.014	0.177	± 0.011
Bed nuclei of the stria terminalis, anterior division, anteromedial area	0.284	± 0.008	0.209	± 0.011
Bed nuclei of the stria terminalis, anterior division, dorsomedial nucleus	0.032	± 0.001	0.024	± 0.002
Bed nuclei of the stria terminalis, anterior division, fusiform nucleus	0.019	± 0.002	0.013	± 0.001
Bed nuclei of the stria terminalis, anterior division, juxtacapsular nucleus	0.011	± 0.002	0.009	± 0.001
Bed nuclei of the stria terminalis, anterior division, magnocellular nucleus	0.058	± 0.001	0.042	± 0.002
Bed nuclei of the stria terminalis, anterior division, oval nucleus	0.062	± 0.003	0.047	± 0.001
Bed nuclei of the stria terminalis, anterior division, rhomboid nucleus	0.044	± 0.003	0.030	± 0.003
Bed nuclei of the stria terminalis, anterior division, ventral nucleus	0.060	± 0.004	0.045	± 0.002
Bed nuclei of the stria terminalis, posterior division	0.491	± 0.008	0.362	± 0.012
Bed nuclei of the stria terminalis, posterior division, dorsal nucleus	0.003	± 0.000	0.003	± 0.000
Bed nuclei of the stria terminalis, posterior division, principal nucleus	0.255	± 0.006	0.191	± 0.007
Bed nuclei of the stria terminalis, posterior division, interfascicular nucleus	0.178	± 0.007	0.130	± 0.004
Bed nuclei of the stria terminalis, posterior division, transverse nucleus	0.032	± 0.002	0.022	± 0.001
Bed nuclei of the stria terminalis, posterior division, strial extension	0.022	± 0.002	0.017	± 0.000
Bed nucleus of the anterior commissure	0.012	± 0.001	0.007	± 0.000
Brain stem	66.229	± 4.275	52.377	± 2.665
Interbrain	22.985	± 0.718	17.652	± 0.747
Thalamus	15.483	± 0.431	11.965	± 0.302
Thalamus, sensory-motor cortex related	4.817	± 0.163	3.737	± 0.112
Ventral group of the dorsal thalamus	3.253	± 0.103	2.484	± 0.070
Ventral anterior-lateral complex of the thalamus	0.928	± 0.034	0.689	± 0.017
Ventral medial nucleus of the thalamus	0.567	± 0.019	0.432	± 0.014
Ventral posterior complex of the thalamus	1.758	± 0.050	1.363	± 0.042
Ventral posterolateral nucleus of the thalamus	0.641	± 0.017	0.506	± 0.017
Ventral posterolateral nucleus of the thalamus, parvicellular part	0.064	± 0.004	0.044	± 0.001
Ventral posteromedial nucleus of the thalamus	0.923	± 0.026	0.717	± 0.024
Ventral posteromedial nucleus of the thalamus, parvicellular part	0.130	± 0.007	0.096	± 0.002
Subparafascicular nucleus	0.276	± 0.008	0.217	± 0.010
Subparafascicular nucleus, magnocellular part	0.065	± 0.004	0.054	± 0.004
Subparafascicular nucleus, parvicellular part	0.211	± 0.006	0.163	± 0.007
Subparafascicular area	0.055	± 0.003	0.043	± 0.003
Peripeduncular nucleus	0.091	± 0.006	0.082	± 0.005
Geniculate group, dorsal thalamus	1.142	± 0.045	0.910	± 0.031
Medial geniculate complex	0.647	± 0.027	0.521	± 0.019
Medial geniculate complex, dorsal part	0.172	± 0.007	0.127	± 0.005
Medial geniculate complex, ventral part	0.347	± 0.018	0.288	± 0.010
Medial geniculate complex, medial part	0.129	± 0.003	0.105	± 0.005
Dorsal part of the lateral geniculate complex	0.495	± 0.021	0.390	± 0.014
Thalamus, polymodal association cortex related	8.785	± 0.234	6.769	± 0.223
Lateral group of the dorsal thalamus	1.691	± 0.051	1.309	± 0.053
Lateral posterior nucleus of the thalamus	0.669	± 0.015	0.531	± 0.025
Posterior complex of the thalamus	0.841	± 0.027	0.636	± 0.027
Posterior limiting nucleus of the thalamus	0.103	± 0.007	0.084	± 0.005
Suprageniculate nucleus	0.077	± 0.004	0.058	± 0.004
Anterior group of the dorsal thalamus	1.606	± 0.042	1.185	± 0.055
Anteroventral nucleus of thalamus	0.285	± 0.007	0.199	± 0.010
Anteromedial nucleus	0.370	± 0.009	0.269	± 0.010
Anteromedial nucleus, dorsal part	0.292	± 0.007	0.214	± 0.009
Anteromedial nucleus, ventral part	0.078	± 0.002	0.055	± 0.001
Anterodorsal nucleus	0.126	± 0.007	0.091	± 0.008
Interanteromedial nucleus of the thalamus	0.037	± 0.002	0.029	± 0.003
Interanterodorsal nucleus of the thalamus	0.067	± 0.003	0.048	± 0.002
Lateral dorsal nucleus of thalamus	0.722	± 0.018	0.549	± 0.027
Medial group of the dorsal thalamus	1.309	± 0.047	1.028	± 0.056
Intermediodorsal nucleus of the thalamus	0.156	± 0.012	0.126	± 0.009
Mediodorsal nucleus of thalamus	0.940	± 0.031	0.742	± 0.047
Mediodorsal nucleus of the thalamus, central part	0.160	± 0.004	0.130	± 0.008
Mediodorsal nucleus of the thalamus, lateral part	0.367	± 0.017	0.274	± 0.019
Mediodorsal nucleus of the thalamus, medial part	0.414	± 0.015	0.338	± 0.023
Submedial nucleus of the thalamus	0.192	± 0.006	0.142	± 0.005

Perireunensis nucleus	0.021	± 0.001	0.017	± 0.001
Midline group of the dorsal thalamus	1.191	± 0.022	0.914	± 0.023
Paraventricular nucleus of the thalamus	0.504	± 0.011	0.382	± 0.005
Parataenial nucleus	0.117	± 0.004	0.091	± 0.003
Nucleus of reunions	0.571	± 0.010	0.441	± 0.019
Intralaminar nuclei of the dorsal thalamus	1.320	± 0.037	1.018	± 0.042
Rhomboid nucleus	0.170	± 0.006	0.134	± 0.008
Central medial nucleus of the thalamus	0.297	± 0.008	0.242	± 0.015
Paracentral nucleus	0.160	± 0.005	0.119	± 0.007
Central lateral nucleus of the thalamus	0.230	± 0.009	0.172	± 0.008
Parafascicular nucleus	0.463	± 0.017	0.351	± 0.007
Reticular nucleus of the thalamus	1.132	± 0.037	0.883	± 0.041
Geniculate group, ventral thalamus	0.092	± 0.003	0.075	± 0.003
Intergeniculate leaflet of the lateral geniculate complex	0.075	± 0.003	0.059	± 0.002
Ventral part of the lateral geniculate complex	0.322	± 0.010	0.267	± 0.012
Subgeniculate nucleus	0.016	± 0.001	0.016	± 0.001
Epithalamus	0.444	± 0.017	0.358	± 0.013
Medial habenula	0.174	± 0.011	0.146	± 0.004
Lateral habenula	0.271	± 0.010	0.213	± 0.010
Hypothalamus	10.649	± 0.398	8.093	± 0.598
Periventricular zone	0.553	± 0.015	0.411	± 0.034
Supraoptic nucleus	0.030	± 0.003	0.021	± 0.002
Accessory supraoptic group	0.001	± 0.000	0.001	± 0.000
Nucleus circularis	0.001	± 0.000	0.001	± 0.000
Paraventricular hypothalamic nucleus	0.215	± 0.002	0.164	± 0.005
Paraventricular hypothalamic nucleus, magnocellular division	0.035	± 0.001	0.031	± 0.001
Paraventricular hypothalamic nucleus, magnocellular division, medial magnocellular part	0.009	± 0.001	0.007	± 0.000
Paraventricular hypothalamic nucleus, magnocellular division, posterior magnocellular part	0.027	± 0.001	0.024	± 0.001
Paraventricular hypothalamic nucleus, magnocellular division, posterior magnocellular part, lateral zon	0.022	± 0.001	0.020	± 0.001
Paraventricular hypothalamic nucleus, magnocellular division, posterior magnocellular part, medial zo	0.005	± 0.001	0.004	± 0.001
Paraventricular hypothalamic nucleus, parvicellular division	0.179	± 0.002	0.132	± 0.005
Paraventricular hypothalamic nucleus, parvicellular division, anterior parvicellular part	0.072	± 0.001	0.054	± 0.002
Paraventricular hypothalamic nucleus, parvicellular division, medial parvicellular part, dorsal zone	0.065	± 0.002	0.047	± 0.004
Paraventricular hypothalamic nucleus, parvicellular division, periventricular part	0.042	± 0.003	0.031	± 0.002
Periventricular hypothalamic nucleus, anterior part	0.013	± 0.001	0.011	± 0.001
Periventricular hypothalamic nucleus, intermediate part	0.094	± 0.002	0.082	± 0.005
Arcuate hypothalamic nucleus	0.199	± 0.011	0.131	± 0.027
Periventricular region	1.809	± 0.059	1.372	± 0.108
Anterodorsal preoptic nucleus	0.027	± 0.002	0.023	± 0.001
Anteroventral preoptic nucleus	0.053	± 0.002	0.043	± 0.004
Anteroventral periventricular nucleus	0.065	± 0.005	0.049	± 0.004
Dorsomedial nucleus of the hypothalamus	0.385	± 0.017	0.297	± 0.026
Dorsomedial nucleus of the hypothalamus, anterior part	0.221	± 0.007	0.182	± 0.013
Dorsomedial nucleus of the hypothalamus, posterior part	0.069	± 0.005	0.049	± 0.004
Dorsomedial nucleus of the hypothalamus, ventral part	0.095	± 0.006	0.066	± 0.011
Median preoptic nucleus	0.133	± 0.005	0.100	± 0.004
Medial preoptic area	0.698	± 0.017	0.537	± 0.031
Vascular organ of the lamina terminalis	0.008	± 0.000	0.007	± 0.001
Posterodorsal preoptic nucleus	0.004	± 0.000	0.003	± 0.001
Parastrial nucleus	0.020	± 0.001	0.014	± 0.001
Periventricular hypothalamic nucleus, posterior part	0.112	± 0.006	0.070	± 0.018
Periventricular hypothalamic nucleus, preoptic part	0.076	± 0.001	0.056	± 0.004
Subparaventricular zone	0.116	± 0.004	0.087	± 0.008
Suprachiasmatic nucleus	0.077	± 0.005	0.058	± 0.009
Subfornical organ	0.012	± 0.001	0.008	± 0.001
Ventrolateral preoptic nucleus	0.023	± 0.001	0.018	± 0.001
Hypothalamic medial zone	2.261	± 0.124	1.746	± 0.118
Anterior hypothalamic nucleus	0.621	± 0.016	0.488	± 0.029
Anterior hypothalamic nucleus, anterior part	0.183	± 0.006	0.142	± 0.010
Anterior hypothalamic nucleus, central part	0.344	± 0.009	0.273	± 0.014
Anterior hypothalamic nucleus, posterior part	0.093	± 0.003	0.073	± 0.004
Mammillary body	0.295	± 0.028	0.233	± 0.019
Lateral mammillary nucleus	0.053	± 0.005	0.039	± 0.004
Medial mammillary nucleus	0.354	± 0.039	0.268	± 0.029
Medial mammillary nucleus, median part	0.010	± 0.002	0.010	± 0.002
Supramammillary nucleus	0.192	± 0.019	0.151	± 0.010
Supramammillary nucleus, lateral part	0.138	± 0.015	0.107	± 0.006
Supramammillary nucleus, medial part	0.054	± 0.004	0.044	± 0.004
Tuberomammillary nucleus	0.040	± 0.003	0.032	± 0.005
Tuberomammillary nucleus, dorsal part	0.011	± 0.000	0.011	± 0.001
Tuberomammillary nucleus, ventral part	0.030	± 0.003	0.022	± 0.004
Medial preoptic nucleus	0.176	± 0.005	0.135	± 0.006
Medial preoptic nucleus, central part	0.004	± 0.000	0.003	± 0.000
Medial preoptic nucleus, lateral part	0.070	± 0.002	0.059	± 0.004
Medial preoptic nucleus, medial part	0.091	± 0.006	0.064	± 0.003
Dorsal premammillary nucleus	0.074	± 0.006	0.057	± 0.004
Ventral premammillary nucleus	0.074	± 0.007	0.047	± 0.010
Paraventricular hypothalamic nucleus, descending division	0.032	± 0.001	0.028	± 0.002
Paraventricular hypothalamic nucleus, descending division, dorsal parvicellular part	0.008	± 0.001	0.007	± 0.001
Paraventricular hypothalamic nucleus, descending division, forniceal part	0.003	± 0.000	0.003	± 0.000
Paraventricular hypothalamic nucleus, descending division, lateral parvicellular part	0.016	± 0.001	0.015	± 0.001
Paraventricular hypothalamic nucleus, descending division, medial parvicellular part, ventral zone	0.004	± 0.001	0.004	± 0.001
Ventromedial hypothalamic nucleus	0.365	± 0.019	0.265	± 0.027
Ventromedial hypothalamic nucleus, anterior part	0.014	± 0.001	0.011	± 0.001
Ventromedial hypothalamic nucleus, central part	0.103	± 0.007	0.073	± 0.009
Ventromedial hypothalamic nucleus, dorsomedial part	0.096	± 0.007	0.069	± 0.006
Ventromedial hypothalamic nucleus, ventrolateral part	0.153	± 0.007	0.112	± 0.012
Posterior hypothalamic nucleus	0.634	± 0.047	0.501	± 0.026
Hypothalamic lateral zone	4.715	± 0.147	3.591	± 0.246
Lateral hypothalamic area	2.987	± 0.088	2.301	± 0.152
Lateral preoptic area	0.887	± 0.018	0.675	± 0.033
Preparasubthalamic nucleus	0.009	± 0.001	0.009	± 0.001
Parasubthalamic nucleus	0.055	± 0.006	0.041	± 0.003
Retrochiasmatic area	0.190	± 0.012	0.136	± 0.021
Subthalamic nucleus	0.164	± 0.007	0.129	± 0.003
Tuberal nucleus	0.357	± 0.016	0.252	± 0.037
Zona incerta	1.572	± 0.046	1.233	± 0.042
Dopaminergic A13 group	0.023	± 0.001	0.015	± 0.001
Fields of Forel	0.043	± 0.003	0.033	± 0.002
Median eminence	0.046	± 0.004	0.027	± 0.008
Midbrain	20.475	± 0.760	15.940	± 0.566
Midbrain, sensory related	6.994	± 0.202	5.492	± 0.166
Superior colliculus, sensory related	1.862	± 0.042	1.516	± 0.046
Superior colliculus, optic layer	0.373	± 0.008	0.302	± 0.013
Superior colliculus, superficial gray layer	0.920	± 0.020	0.748	± 0.022
Superior colliculus, zonal layer	0.569	± 0.016	0.465	± 0.014
Inferior colliculus	4.803	± 0.154	3.723	± 0.115
Inferior colliculus, central nucleus	1.359	± 0.030	1.049	± 0.033

Inferior colliculus, dorsal nucleus	0.889	± 0.051	0.699	± 0.043
Inferior colliculus, external nucleus	2.555	± 0.078	1.975	± 0.056
Nucleus of the brachium of the inferior colliculus	0.107	± 0.009	0.083	± 0.007
Nucleus sagulum	0.079	± 0.005	0.059	± 0.003
Parabigeminal nucleus	0.063	± 0.007	0.050	± 0.005
Midbrain trigeminal nucleus	0.080	± 0.002	0.062	± 0.005
Midbrain, motor related	8.598	± 0.329	6.629	± 0.246
Substantia nigra, reticular part	1.107	± 0.096	0.881	± 0.077
Ventral tegmental area	0.743	± 0.062	0.590	± 0.042
Midbrain reticular nucleus, retrorubral area	0.322	± 0.013	0.255	± 0.015
Midbrain reticular nucleus	4.170	± 0.167	3.218	± 0.135
Superior colliculus, motor related	4.310	± 0.118	3.264	± 0.092
Superior colliculus, motor related, deep gray layer	1.883	± 0.049	1.432	± 0.042
Superior colliculus, motor related, deep white layer	0.087	± 0.002	0.066	± 0.003
Superior colliculus, motor related, intermediate white layer	0.733	± 0.029	0.561	± 0.022
Superior colliculus, motor related, intermediate gray layer	2.750	± 0.059	2.095	± 0.059
Superior colliculus, motor related, intermediate gray layer, sublayer a	0.430	± 0.011	0.322	± 0.006
Superior colliculus, motor related, intermediate gray layer, sublayer b	0.943	± 0.031	0.702	± 0.023
Superior colliculus, motor related, intermediate gray layer, sublayer c	0.234	± 0.006	0.181	± 0.005
Periaqueductal gray	4.185	± 0.114	3.232	± 0.077
Precommissural nucleus	0.061	± 0.003	0.048	± 0.002
Interstitial nucleus of Cajal	0.036	± 0.002	0.028	± 0.001
Nucleus of Darkschewitsch	0.065	± 0.004	0.051	± 0.002
Pretectal region	1.270	± 0.026	0.982	± 0.026
Anterior pretectal nucleus	0.731	± 0.016	0.557	± 0.019
Medial pretectal area	0.086	± 0.004	0.072	± 0.002
Nucleus of the optic tract	0.128	± 0.002	0.099	± 0.004
Nucleus of the posterior commissure	0.212	± 0.007	0.162	± 0.005
Olivary pretectal nucleus	0.065	± 0.001	0.051	± 0.002
Posterior pretectal nucleus	0.049	± 0.001	0.040	± 0.001
Cuneiform nucleus	0.279	± 0.007	0.214	± 0.008
Red nucleus	0.269	± 0.015	0.211	± 0.011
Oculomotor nucleus	0.037	± 0.001	0.025	± 0.002
Edinger-Westphal nucleus	0.026	± 0.002	0.019	± 0.001
Trochlear nucleus	0.011	± 0.001	0.008	± 0.000
Ventral tegmental nucleus	0.035	± 0.002	0.031	± 0.002
Anterior tegmental nucleus	0.019	± 0.002	0.016	± 0.001
Lateral terminal nucleus of the accessory optic tract	0.009	± 0.001	0.006	± 0.000
Midbrain, behavioral state related	1.270	± 0.067	1.028	± 0.060
Substantia nigra, compact part	0.340	± 0.023	0.279	± 0.020
Pedunculopontine nucleus	0.366	± 0.015	0.289	± 0.017
Midbrain raphe nuclei	0.565	± 0.032	0.461	± 0.025
Interfascicular nucleus raphe	0.035	± 0.003	0.027	± 0.002
Interpeduncular nucleus	0.271	± 0.023	0.215	± 0.015
Rostral linear nucleus raphe	0.033	± 0.003	0.027	± 0.002
Central linear nucleus raphe	0.087	± 0.005	0.074	± 0.004
Dorsal nucleus raphe	0.138	± 0.006	0.117	± 0.004
Hindbrain	26.381	± 3.490	21.576	± 1.776
Pons	14.881	± 0.716	11.955	± 0.620
Pons, sensory related	2.998	± 0.200	2.386	± 0.144
Nucleus of the lateral lemniscus	0.782	± 0.064	0.618	± 0.046
Nucleus of the lateral lemniscus, dorsal part	0.200	± 0.014	0.153	± 0.007
Nucleus of the lateral lemniscus, horizontal part	0.052	± 0.004	0.040	± 0.002
Nucleus of the lateral lemniscus, ventral part	0.530	± 0.047	0.425	± 0.037
Principal sensory nucleus of the trigeminal	0.823	± 0.069	0.684	± 0.040
Parabrachial nucleus	0.751	± 0.023	0.581	± 0.027
Koelliker-Fuse subnucleus	0.125	± 0.007	0.096	± 0.005
Parabrachial nucleus, lateral division	0.420	± 0.012	0.322	± 0.014
Parabrachial nucleus, lateral division, central lateral part	0.133	± 0.003	0.100	± 0.003
Parabrachial nucleus, lateral division, dorsal lateral part	0.058	± 0.002	0.043	± 0.002
Parabrachial nucleus, lateral division, external lateral part	0.063	± 0.003	0.051	± 0.005
Parabrachial nucleus, lateral division, superior lateral part	0.088	± 0.003	0.067	± 0.003
Parabrachial nucleus, lateral division, ventral lateral part	0.078	± 0.003	0.061	± 0.003
Parabrachial nucleus, medial division	0.206	± 0.004	0.164	± 0.008
Parabrachial nucleus, medial division, external medial part	0.022	± 0.002	0.017	± 0.001
Parabrachial nucleus, medial division, medial medial part	0.184	± 0.003	0.147	± 0.008
Superior olivary complex	0.642	± 0.065	0.503	± 0.032
Superior olivary complex, periolivary region	0.547	± 0.051	0.422	± 0.027
Superior olivary complex, medial part	0.040	± 0.002	0.030	± 0.002
Superior olivary complex, lateral part	0.055	± 0.014	0.050	± 0.003
Pons, motor related	4.646	± 0.206	3.679	± 0.198
Barrington's nucleus	0.027	± 0.002	0.023	± 0.001
Dorsal tegmental nucleus	0.085	± 0.004	0.072	± 0.004
Pontine central gray	0.580	± 0.009	0.445	± 0.023
Pontine gray	0.688	± 0.073	0.498	± 0.048
Pontine reticular nucleus, caudal part	2.243	± 0.127	1.840	± 0.084
Supragenual nucleus	0.012	± 0.001	0.010	± 0.001
Supratrigeminal nucleus	0.123	± 0.003	0.101	± 0.006
Tegmental reticular nucleus	0.521	± 0.032	0.401	± 0.024
Motor nucleus of trigeminal	0.368	± 0.008	0.290	± 0.017
Pons, behavioral state related	2.978	± 0.099	2.319	± 0.135
Superior central nucleus raphe	0.434	± 0.014	0.345	± 0.015
Superior central nucleus raphe, lateral part	0.233	± 0.011	0.182	± 0.009
Superior central nucleus raphe, medial part	0.200	± 0.005	0.162	± 0.007
Locus ceruleus	0.045	± 0.000	0.037	± 0.003
Laterodorsal tegmental nucleus	0.117	± 0.001	0.094	± 0.004
Nucleus incertus	0.106	± 0.003	0.083	± 0.006
Pontine reticular nucleus	2.142	± 0.082	1.646	± 0.103
Nucleus raphe pontis	0.044	± 0.001	0.040	± 0.002
Subceruleus nucleus	0.025	± 0.001	0.018	± 0.001
Sublaterodorsal nucleus	0.067	± 0.003	0.056	± 0.004
Medulla	20.033	± 4.062	17.001	± 1.788
Medulla, sensory related	4.486	± 0.967	3.783	± 0.402
Area postrema	0.025	± 0.009	0.023	± 0.004
Cochlear nuclei	1.270	± 0.071	1.029	± 0.047
Granular lamina of the cochlear nuclei	0.035	± 0.001	0.029	± 0.002
Cochlear nucleus, subpedunclular granular region	0.034	± 0.001	0.027	± 0.002
Dorsal cochlear nucleus	0.461	± 0.013	0.364	± 0.022
Ventral cochlear nucleus	0.740	± 0.062	0.608	± 0.022
Dorsal column nuclei	0.177	± 0.058	0.150	± 0.027
Cuneate nucleus	0.138	± 0.044	0.126	± 0.019
Gracile nucleus	0.039	± 0.014	0.024	± 0.008
External cuneate nucleus	0.211	± 0.052	0.180	± 0.029
Nucleus of the trapezoid body	0.117	± 0.013	0.082	± 0.003
Nucleus of the solitary tract	0.545	± 0.117	0.466	± 0.037
Nucleus of the solitary tract, central part	0.012	± 0.003	0.010	± 0.002
Nucleus of the solitary tract, commissural part	0.040	± 0.014	0.035	± 0.005
Nucleus of the solitary tract, gelatinous part	0.015	± 0.004	0.014	± 0.001

Nucleus of the solitary tract, lateral part	0.212	± 0.051	0.175	± 0.014
Nucleus of the solitary tract, medial part	0.266	± 0.046	0.231	± 0.017
Spinal nucleus of the trigeminal, caudal part	0.606	± 0.218	0.411	± 0.147
Spinal nucleus of the trigeminal, interpolar part	1.146	± 0.379	1.055	± 0.143
Spinal nucleus of the trigeminal, oral part	0.389	± 0.088	0.388	± 0.013
Spinal nucleus of the trigeminal, oral part, caudal dorsomedial part	0.029	± 0.004	0.027	± 0.001
Spinal nucleus of the trigeminal, oral part, middle dorsomedial part, dorsal zone	0.031	± 0.004	0.031	± 0.001
Spinal nucleus of the trigeminal, oral part, middle dorsomedial part, ventral zone	0.039	± 0.009	0.037	± 0.002
Spinal nucleus of the trigeminal, oral part, rostral dorsomedial part	0.070	± 0.004	0.058	± 0.003
Spinal nucleus of the trigeminal, oral part, ventrolateral part	0.221	± 0.067	0.234	± 0.008
Medulla, motor related	11.122	± 2.122	9.280	± 1.053
Abducens nucleus	0.013	± 0.000	0.010	± 0.001
Facial motor nucleus	0.411	± 0.137	0.432	± 0.011
Accessory facial motor nucleus	0.011	± 0.002	0.010	± 0.001
Nucleus ambiguus	0.088	± 0.030	0.072	± 0.017
Nucleus ambiguus, dorsal division	0.017	± 0.006	0.013	± 0.003
Nucleus ambiguus, ventral division	0.045	± 0.015	0.039	± 0.006
Dorsal motor nucleus of the vagus nerve	0.086	± 0.028	0.067	± 0.009
Gigantocellular reticular nucleus	1.959	± 0.389	1.732	± 0.171
Infracerebellar nucleus	0.008	± 0.001	0.006	± 0.001
Inferior olivary complex	0.246	± 0.083	0.212	± 0.069
Intermediate reticular nucleus	1.888	± 0.427	1.547	± 0.225
Inferior salivatory nucleus	0.040	± 0.002	0.034	± 0.001
Linear nucleus of the medulla	0.023	± 0.008	0.020	± 0.004
Lateral reticular nucleus	0.347	± 0.118	0.250	± 0.091
Lateral reticular nucleus, magnocellular part	0.312	± 0.106	0.226	± 0.083
Lateral reticular nucleus, parvicellular part	0.028	± 0.009	0.019	± 0.006
Magnocellular reticular nucleus	0.433	± 0.141	0.400	± 0.047
Medullary reticular nucleus	1.088	± 0.384	0.759	± 0.303
Medullary reticular nucleus, dorsal part	0.359	± 0.127	0.234	± 0.090
Medullary reticular nucleus, ventral part	0.729	± 0.257	0.524	± 0.213
Parvicellular reticular nucleus	1.106	± 0.164	0.962	± 0.050
Parasolitary nucleus	0.022	± 0.008	0.018	± 0.002
Paragigantocellular reticular nucleus	0.624	± 0.144	0.546	± 0.048
Paragigantocellular reticular nucleus, dorsal part	0.196	± 0.008	0.160	± 0.006
Paragigantocellular reticular nucleus, lateral part	0.428	± 0.143	0.386	± 0.046
Perihypoglossal nuclei	0.348	± 0.012	0.270	± 0.016
Nucleus of Roller	0.028	± 0.002	0.020	± 0.002
Nucleus prepositus	0.319	± 0.012	0.250	± 0.015
Parapyramidal nucleus	0.028	± 0.009	0.037	± 0.002
Vestibular nuclei	2.090	± 0.060	1.691	± 0.072
Lateral vestibular nucleus	0.351	± 0.010	0.288	± 0.014
Medial vestibular nucleus	1.050	± 0.031	0.836	± 0.039
Spinal vestibular nucleus	0.407	± 0.018	0.348	± 0.017
Superior vestibular nucleus	0.282	± 0.005	0.219	± 0.007
Nucleus x	0.037	± 0.004	0.033	± 0.002
Hypoglossal nucleus	0.268	± 0.071	0.218	± 0.035
Nucleus y	0.019	± 0.000	0.015	± 0.001
Medulla, behavioral state related	0.151	± 0.035	0.130	± 0.021
Nucleus raphe magnus	0.082	± 0.012	0.073	± 0.005
Nucleus raphe pallidus	0.034	± 0.011	0.029	± 0.008
Nucleus raphe obscurus	0.035	± 0.012	0.028	± 0.009
Cerebellum	43.744	± 2.190	33.142	± 2.214
Cerebellar cortex	36.851	± 2.038	28.073	± 1.880
Vermal regions	14.901	± 0.999	11.993	± 0.413
Lingula (I)	0.103	± 0.004	0.076	± 0.005
Lingula (I), molecular layer	0.060	± 0.002	0.045	± 0.002
Lingula (I), granular layer	0.043	± 0.003	0.031	± 0.003
Central lobule	3.423	± 0.020	2.567	± 0.091
Lobule II	1.159	± 0.012	0.890	± 0.031
Lobule II, molecular layer	0.621	± 0.007	0.483	± 0.014
Lobule II, granular layer	0.538	± 0.006	0.407	± 0.018
Lobule III	2.263	± 0.019	1.676	± 0.062
Lobule III, molecular layer	1.190	± 0.014	0.886	± 0.026
Lobule III, granular layer	1.073	± 0.011	0.791	± 0.036
Culmen	5.867	± 0.167	4.615	± 0.103
Lobules IV-V	5.867	± 0.167	4.615	± 0.103
Lobules IV-V, molecular layer	3.188	± 0.091	2.513	± 0.063
Lobules IV-V, granular layer	2.679	± 0.080	2.102	± 0.041
Declive (VI)	1.793	± 0.340	1.791	± 0.044
Declive (VI), molecular layer	0.904	± 0.162	0.916	± 0.023
Declive (VI), granular layer	0.890	± 0.179	0.875	± 0.026
Folium-tuber vermis (VII)	0.284	± 0.087	0.277	± 0.026
Folium-tuber vermis (VII), molecular layer	0.155	± 0.047	0.148	± 0.017
Folium-tuber vermis (VII), granular layer	0.129	± 0.041	0.129	± 0.009
Pyramus (VIII)	0.579	± 0.160	0.395	± 0.131
Pyramus (VIII), molecular layer	0.276	± 0.081	0.193	± 0.063
Pyramus (VIII), granular layer	0.303	± 0.079	0.202	± 0.067
Uvula (IX)	1.536	± 0.353	1.215	± 0.153
Uvula (IX), molecular layer	0.804	± 0.179	0.631	± 0.080
Uvula (IX), granular layer	0.732	± 0.174	0.584	± 0.073
Nodulus (X)	1.317	± 0.056	1.058	± 0.063
Nodulus (X), molecular layer	0.659	± 0.034	0.538	± 0.035
Nodulus (X), granular layer	0.658	± 0.024	0.520	± 0.027
Hemispheric regions	21.950	± 1.240	16.080	± 1.468
Simple lobule	4.615	± 0.156	3.487	± 0.155
Simple lobule, molecular layer	2.596	± 0.088	1.965	± 0.088
Simple lobule, granular layer	2.019	± 0.068	1.522	± 0.067
Ansiform lobule	7.896	± 0.441	5.708	± 0.499
Crus 1	4.403	± 0.231	3.265	± 0.237
Crus 1, molecular layer	2.371	± 0.122	1.770	± 0.128
Crus 1, granular layer	2.032	± 0.116	1.495	± 0.110
Crus 2	3.492	± 0.215	2.442	± 0.264
Crus 2, molecular layer	1.807	± 0.117	1.267	± 0.131
Crus 2, granular layer	1.685	± 0.105	1.176	± 0.135
Paramedian lobule	3.017	± 0.213	2.123	± 0.321
Paramedian lobule, molecular layer	1.662	± 0.124	1.185	± 0.181
Paramedian lobule, granular layer	1.355	± 0.092	0.938	± 0.141
Copula pyramidis	1.581	± 0.255	1.128	± 0.260
Copula pyramidis, molecular layer	0.814	± 0.122	0.584	± 0.130
Copula pyramidis, granular layer	0.768	± 0.133	0.543	± 0.130
Paraflocculus	3.634	± 0.309	2.655	± 0.448
Paraflocculus, molecular layer	1.855	± 0.173	1.361	± 0.219
Paraflocculus, granular layer	1.778	± 0.139	1.294	± 0.231
Flocculus	1.207	± 0.055	0.980	± 0.038
Flocculus, molecular layer	0.626	± 0.029	0.504	± 0.021
Flocculus, granular layer	0.581	± 0.026	0.475	± 0.017
Cerebellar nuclei	1.048	± 0.021	0.762	± 0.062

Fastigial nucleus	0.365	± 0.009	0.253	± 0.026
Interposed nucleus	0.455	± 0.008	0.334	± 0.027
Dentate nucleus	0.228	± 0.006	0.175	± 0.011
fiber tracts	30.071	± 2.050	25.949	± 2.325
cranial nerves	9.536	± 0.751	8.318	± 0.933
vomeronasal nerve	0.024	± 0.003	0.022	± 0.006
olfactory nerve	3.243	± 0.357	2.991	± 0.386
olfactory nerve layer of main olfactory bulb	1.519	± 0.171	1.304	± 0.116
lateral olfactory tract, general	0.753	± 0.101	0.703	± 0.161
lateral olfactory tract, body	0.742	± 0.098	0.694	± 0.159
dorsal limb	0.011	± 0.003	0.009	± 0.002
anterior commissure, olfactory limb	0.971	± 0.139	0.984	± 0.195
optic nerve	1.252	± 0.112	1.085	± 0.110
brachium of the superior colliculus	0.322	± 0.014	0.289	± 0.016
superior colliculus commissure	0.024	± 0.002	0.019	± 0.001
optic chiasm	0.148	± 0.031	0.123	± 0.034
optic tract	0.758	± 0.080	0.653	± 0.071
oculomotor nerve	0.938	± 0.065	0.828	± 0.054
medial longitudinal fascicle	0.903	± 0.063	0.800	± 0.054
trigeminal nerve	1.376	± 0.134	1.156	± 0.124
motor root of the trigeminal nerve	0.161	± 0.020	0.112	± 0.036
sensory root of the trigeminal nerve	1.118	± 0.106	0.965	± 0.089
midbrain tract of the trigeminal nerve	0.015	± 0.001	0.010	± 0.003
spinal tract of the trigeminal nerve	1.103	± 0.104	0.955	± 0.088
facial nerve	0.217	± 0.031	0.161	± 0.053
genu of the facial nerve	0.086	± 0.011	0.062	± 0.023
vestibulocochlear nerve	1.839	± 0.122	1.430	± 0.244
vestibular nerve	0.132	± 0.022	0.116	± 0.032
cochlear nerve	1.674	± 0.107	1.284	± 0.218
trapezoid body	0.327	± 0.039	0.286	± 0.041
lateral lemniscus	0.991	± 0.071	0.709	± 0.158
inferior colliculus commissure	0.003	± 0.001	0.002	± 0.001
brachium of the inferior colliculus	0.353	± 0.011	0.287	± 0.040
vagus nerve	0.039	± 0.005	0.026	± 0.003
solitary tract	0.039	± 0.005	0.026	± 0.003
dorsal roots	0.870	± 0.041	0.827	± 0.078
cervicothalamic tract	0.870	± 0.041	0.827	± 0.078
dorsal column	0.027	± 0.009	0.030	± 0.011
cuneate fascicle	0.023	± 0.008	0.025	± 0.010
gracile fascicle	0.005	± 0.002	0.005	± 0.002
medial lemniscus	0.842	± 0.039	0.797	± 0.081
cerebellum related fiber tracts	8.350	± 0.883	6.552	± 0.924
cerebellar commissure	0.292	± 0.019	0.260	± 0.040
cerebellar peduncles	2.313	± 0.160	2.047	± 0.244
superior cerebelar peduncles	1.254	± 0.054	1.119	± 0.122
superior cerebellar peduncle decussation	0.208	± 0.006	0.141	± 0.036
ventral spinocerebellar tract	0.832	± 0.039	0.810	± 0.081
middle cerebellar peduncle	1.481	± 0.125	1.237	± 0.203
inferior cerebellar peduncle	0.724	± 0.096	0.618	± 0.089
arbor vitae	5.745	± 0.761	4.246	± 0.666
lateral forebrain bundle system	6.433	± 0.263	5.758	± 0.401
corpus callosum	5.221	± 0.264	4.654	± 0.553
corpus callosum, anterior forceps	2.369	± 0.059	2.105	± 0.154
external capsule	1.651	± 0.060	1.366	± 0.103
genu of corpus callosum	0.394	± 0.014	0.395	± 0.053
corpus callosum, posterior forceps	0.345	± 0.051	0.285	± 0.032
corpus callosum, splenium	0.109	± 0.016	0.094	± 0.015
corticospinal tract	3.878	± 0.213	3.542	± 0.234
internal capsule	1.869	± 0.055	1.798	± 0.165
cerebal peduncle	1.675	± 0.137	1.456	± 0.091
pyramid	0.187	± 0.031	0.167	± 0.012
thalamus related	0.203	± 0.009	0.198	± 0.016
external medullary lamina of the thalamus	0.191	± 0.009	0.185	± 0.015
internal medullary lamina of the thalamus	0.012	± 0.001	0.012	± 0.002
extrapyramidal fiber systems	0.102	± 0.010	0.082	± 0.009
tectospinal pathway	0.189	± 0.009	0.125	± 0.031
doral tegmental decussation	0.032	± 0.002	0.023	± 0.004
rubrospinal tract	0.386	± 0.020	0.332	± 0.036
ventral tegmental decussation	0.069	± 0.008	0.059	± 0.006
medial forebrain bundle system	5.650	± 0.270	5.238	± 0.572
cerebrum related	4.886	± 0.258	4.509	± 0.494
amygdalar capsule	0.153	± 0.005	0.120	± 0.010
anterior commissure, temporal limb	0.150	± 0.007	0.127	± 0.011
cingulum bundle	0.430	± 0.059	0.392	± 0.074
fornix system	3.567	± 0.202	3.318	± 0.374
alveus	1.190	± 0.098	1.017	± 0.101
dorsal fornix	0.101	± 0.018	0.096	± 0.021
fimbria	1.272	± 0.014	1.277	± 0.152
postcommissural fornix	0.272	± 0.013	0.255	± 0.030
columns of the fornix	0.272	± 0.013	0.255	± 0.030
hippocampal commissures	0.732	± 0.063	0.674	± 0.078
dorsal hippocampal commissure	0.593	± 0.055	0.510	± 0.046
ventral hippocampal commissure	0.140	± 0.009	0.163	± 0.034
stria terminalis	0.585	± 0.018	0.551	± 0.042
hypothalamus related	0.764	± 0.030	0.730	± 0.084
supramammillary decussation	0.019	± 0.002	0.018	± 0.001
mammillary related	0.210	± 0.017	0.180	± 0.012
principal mammillary tract	0.015	± 0.003	0.014	± 0.002
mammilothalmic tract	0.102	± 0.007	0.089	± 0.007
mammillotegmental tract	0.069	± 0.007	0.057	± 0.004
mammillary peduncle	0.024	± 0.003	0.020	± 0.002
epithalamus related	0.534	± 0.012	0.531	± 0.081
stria medullaris	0.321	± 0.004	0.329	± 0.058
fasciculus retroflexus	0.184	± 0.012	0.178	± 0.020
habenular commissure	0.029	± 0.002	0.024	± 0.004

n = 4 mice per group.

Supplementary Table 3. Left hemisphere dominant regions in *Mecp2*-KO mice.

Left hemisphere dominant regions in *Mecp2*-KO mice		
Region	LI	
	WT	*Mecp2*-KO
Nucleus of the lateral olfactory tract	0.07 ± 3.20	24.25 ± 4.01
Nucleus of the lateral olfactory tract, molecular layer	-0.72 ± 3.28	26.50 ± 4.63
Nucleus of the lateral olfactory tract, pyramidal layer	0.40 ± 2.92	24.16 ± 5.34
Cortical amygdalar area, anterior part, layer 1	-3.36 ± 2.61	22.87 ± 5.18
Entorhinal area, medial part, dorsal zone, layer 2a	3.77 ± 3.67	32.60 ± 5.43
Posterodorsal preoptic nucleus	11.11 ± 7.86	32.74 ± 6.72
Median eminence	-18.84 ± 6.15	25.78 ± 9.33
Oculomotor nucleus	4.19 ± 5.74	22.53 ± 5.01
Ventral tegmental nucleus	4.11 ± 1.77	29.38 ± 4.02
Midbrain raphe nuclei	5.47 ± 8.75	30.35 ± 12.80
Rostral linear nucleus raphe	2.54 ± 1.35	36.99 ± 5.67
Central linear nucleus raphe	-3.15 ± 3.86	34.04 ± 7.36
Dorsal tegmental nucleus	-8.75 ± 3.31	39.03 ± 13.12
Pontine central gray	-0.71 ± 1.80	20.32 ± 4.45
Supragenual nucleus	-4.00 ± 8.38	36.29 ± 7.00
Superior central nucleus raphe	-1.53 ± 2.35	34.54 ± 7.99
Superior central nucleus raphe, lateral part	-1.04 ± 4.53	20.28 ± 7.17
Superior central nucleus raphe, medial part	-1.85 ± 1.80	50.19 ± 10.64
Nucleus incertus	-1.93 ± 9.25	40.47 ± 8.49
Nucleus raphe pontis	-10.51 ± 6.36	42.70 ± 13.65
Area postrema	16.87 ± 2.62	61.12 ± 12.81
Gracile nucleus	-8.85 ± 3.35	24.79 ± 5.36
Nucleus of the solitary tract	7.18 ± 7.32	20.74 ± 8.70
Nucleus of the solitary tract, central part	15.83 ± 5.95	32.19 ± 9.54
Nucleus of the solitary tract, commissural part	-1.25 ± 0.09	46.53 ± 13.81
Nucleus of the solitary tract, gelatinous part	-1.75 ± 1.13	37.63 ± 10.91
Nucleus of the solitary tract, medial part	2.41 ± 3.27	22.29 ± 4.98
Spinal nucleus of the trigeminal, caudal part	-1.08 ± 0.27	30.62 ± 9.99
Nucleus ambiguus	4.58 ± 0.79	24.01 ± 3.90
Nucleus ambiguus, dorsal division	7.64 ± 7.40	36.81 ± 12.20
Dorsal motor nucleus of the vagus nerve	2.48 ± 3.47	35.68 ± 11.93
Gigantocellular reticular nucleus	-3.77 ± 1.70	30.86 ± 8.48
Inferior olivary complex	-0.15 ± 0.95	52.05 ± 7.69
Lateral reticular nucleus	-0.31 ± 0.55	46.38 ± 7.57
Lateral reticular nucleus, magnocellular part	0.60 ± 0.28	47.04 ± 9.43
Lateral reticular nucleus, parvicellular part	-8.88 ± 3.85	36.58 ± 10.75
Magnocellular reticular nucleus	7.23 ± 4.48	33.82 ± 13.69
Paragigantocellular reticular nucleus	-8.88 ± 7.55	26.86 ± 9.37
Paragigantocellular reticular nucleus, dorsal part	-4.81 ± 4.08	35.63 ± 4.32
Paragigantocellular reticular nucleus, lateral part	-3.36 ± 1.72	22.83 ± 3.43
Perihypoglossal nuclei	-8.04 ± 9.51	37.65 ± 13.43
Nucleus of Roller	-8.60 ± 4.33	33.70 ± 6.62
Nucleus prepositus	-7.88 ± 3.30	38.02 ± 8.17
Parapyramidal nucleus	1.70 ± 0.31	33.70 ± 6.18
Hypoglossal nucleus	-8.26 ± 4.12	44.59 ± 4.25
Medulla, behavioral state related	-5.81 ± 1.42	67.68 ± 18.32
Nucleus raphe magnus	-2.20 ± 2.64	71.74 ± 15.91
Nucleus raphe pallidus	-0.43 ± 3.22	67.66 ± 19.00
Nucleus raphe obscurus	8.89 ± 0.82	60.09 ± 4.33
Lingula (I)	-0.56 ± 5.51	37.04 ± 4.33
Lingula (I), molecular layer	-0.05 ± 6.23	40.01 ± 4.29
Lingula (I), granular layer	-1.35 ± 4.66	32.76 ± 5.04
Central lobule	3.42 ± 1.90	21.10 ± 2.77

Lobule II	1.86	± 2.11	22.46	± 6.36
Lobule II, molecular layer	1.10	± 3.67	22.95	± 6.40
Lobule II, granular layer	2.71	± 1.67	21.86	± 6.37
Lobule III	4.23	± 1.82	20.27	± 5.90
Lobule III, granular layer	3.08	± 1.71	20.77	± 5.81
Declive (VI), molecular layer	0.07	± 3.56	20.42	± 3.70
Folium-tuber vermis (VII)	-16.74	± 4.24	32.54	± 9.61
Folium-tuber vermis (VII), molecular layer	-13.58	± 4.02	30.54	± 7.06
Pyramus (VIII)	-3.46	± 7.79	22.11	± 5.12
Pyramus (VIII), granular layer	-3.38	± 4.77	35.04	± 3.70
Uvula (IX)	2.25	± 1.73	32.97	± 7.02
Uvula (IX), molecular layer	2.25	± 1.23	31.25	± 11.12
Uvula (IX), granular layer	2.17	± 2.40	34.89	± 11.15
Nodulus (X)	-0.40	± 3.61	25.93	± 6.79
Nodulus (X), molecular layer	-0.54	± 2.41	25.15	± 7.63
Nodulus (X), granular layer	-0.20	± 4.87	26.76	± 5.98

n = 4 mice per group.

Supplementary Table 4. Right hemisphere dominant regions in *Mecp2*-KO mice.

Right hemisphere dominant regions in *Mecp2*-KO mice

Region	LI	
	WT	*Mecp2*-KO
Frontal pole	1.18 ± 1.08	-22.56 ± 2.01
Frontal pole, layer 1	0.60 ± 1.05	-22.26 ± 4.46
Frontal pole, layer 2/3	4.10 ± 1.24	-24.11 ± 2.10
Anterior cingulate area, ventral part, layer 1	10.76 ± 7.08	-28.95 ± 8.80
Prelimbic area, layer 1	-1.28 ± 3.00	-49.03 ± 9.43
Prelimbic area, layer 2	4.05 ± 4.49	-29.91 ± 2.06
Prelimbic area, layer 2/3	3.06 ± 2.26	-22.78 ± 5.45
Infralimbic area, layer 1	2.86 ± 1.92	-44.12 ± 6.51
Orbital area, medial part	0.10 ± 1.63	-27.55 ± 9.34
Orbital area, medial part, layer 1	-6.10 ± 3.81	-51.25 ± 16.12
Orbital area, medial part, layer 2	5.99 ± 4.78	-31.02 ± 8.14
Orbital area, medial part, layer 2/3	4.73 ± 3.99	-33.29 ± 3.18
Main olfactory bulb	-6.45 ± 5.34	-29.23 ± 8.53
Main olfactory bulb, glomerular layer	-8.12 ± 4.47	-41.77 ± 12.81
Main olfactory bulb, inner plexiform layer	-8.08 ± 4.91	-26.34 ± 3.07
Main olfactory bulb, mitral layer	-6.54 ± 3.96	-28.49 ± 6.64
Main olfactory bulb, outer plexiform layer	-7.29 ± 5.85	-33.72 ± 6.51
Induseum griseum	-5.66 ± 4.70	-30.20 ± 8.51
Medial septal nucleus	-11.03 ± 6.95	-28.84 ± 3.63
Subfornical organ	14.92 ± 3.99	-33.48 ± 10.12

n = 4 mice per group.
